# Adenosine receptors differentially mediate enteric glial cell death induced by *Clostridioides difficile* Toxins A and B

**DOI:** 10.3389/fimmu.2022.956326

**Published:** 2023-01-16

**Authors:** Deiziane V. S. Costa, Jae H. Shin, Sophia M. Goldbeck, David T. Bolick, Flavio S. Mesquita, Andrea V. Loureiro, Mônica J. Rodrigues-Jesus, Gerly A. C. Brito, Cirle A. Warren

**Affiliations:** ^1^ Division of Infectious Diseases and International Health, University of Virginia, Charlottesville, VA, United States; ^2^ Department of Microbiology, University of Sao Paulo, Sao Paulo, Brazil; ^3^ Department of Morphology, Faculty of Medicine, Federal University of Ceará, Fortaleza, Ceará, Brazil

**Keywords:** *Clostridioides difficile*, *Clostridioides difficile* infection, adenosine receptors, enteric glia, cell death

## Abstract

Increased risk of intestinal dysfunction has been reported in patients after *Clostridioides difficile* infection (CDI). Enteric glial cells (EGCs), a component of the enteric nervous system (ENS), contribute to gut homeostasis. Previous studies showed that adenosine receptors, A2A and A2B, modulate inflammation during CDI. However, it is unknown how these receptors can modulate the EGC response to the *C. difficile* toxins (TcdA and TcdB). We investigated the effects of these toxins on the expression of adenosine receptors in EGCs and the role of these receptors on toxin-induced EGC death. Rat EGCs line were incubated with TcdA or TcdB alone or in combination with adenosine analogues 1h prior to toxins challenge. After incubation, EGCs were collected to evaluate gene expression (adenosine receptors and proinflammatory markers) and cell death. *In vivo*, WT, A2A, and A2B KO mice were infected with *C. difficile*, euthanized on day 3 post-infection, and cecum tissue was processed. TcdA and TcdB increased A2A and A3 transcripts, as well as decreased A2B. A2A agonist, but not A2A antagonist, decreased apoptosis induced by TcdA and TcdB in EGCs. A2B blocker, but not A2B agonist, diminished apoptosis in EGCs challenged with both toxins. A3 agonist, but not A3 blocker, reduced apoptosis in EGCs challenged with TcdA and TcdB. Inhibition of protein kinase A (PKA) and CREB, both involved in the main signaling pathway driven by activation of adenosine receptors, decreased EGC apoptosis induced by both toxins. A2A agonist and A2B antagonist decreased S100B upregulation induced by *C. difficile* toxins in EGCs. *In vivo*, infected A2B KO mice, but not A2A, exhibited a decrease in cell death, including EGCs and enteric neuron loss, compared to infected WT mice, reduced intestinal damage and decreased IL-6 and S100B levels in cecum. Our findings indicate that upregulation of A2A and A3 and downregulation of A2B in EGCs and downregulation of A2B in intestinal tissues elicit a protective response against C. *difficile* toxins. Adenosine receptors appear to play a regulatory role in EGCs death and proinflammatory response induced by TcdA and TcdB, and thus may be potential targets of intervention to prevent post-CDI intestinal dysmotility.

## Introduction

*Clostridioides difficile* (*C. difficile*) is an anaerobic, gram-positive, spore-forming bacterium which is transmitted by oral ingestion of spores. Once the microbiota is disrupted in the gut, these spores can germinate and grow into vegetative cells, which in turn secrete toxins (1). *C. difficile* toxins A (TcdA) and B (TcdB) are the main virulence factors involved in *C. difficile* infection (CDI), promoting gut cell death and inflammation (2–5).

Severe CDI has been associated with older age, previous exposure to antibiotics or antacid drugs, multiple comorbidities (such as cardiovascular and neurological diseases, diabetes, hypertension, obesity, and being immunocompromised) (6, 7). CDI treatment involves administration of either vancomycin or fidaxomicin. Alternative options for recurrent CDI include fecal microbiota transplantation and anti-toxin antibodies. However, some patients are not responsive to current standard of care or alternative treatments (8). Given that the immune response of the individual during CDI can potentially contribute to the success of the current anti-*C. difficile* therapeutics, understanding the pathogenesis of CDI might be the key to the development of adjunctive treatments able to modulate this immune response.

The pathogenesis of CDI involves several pathways, including adenosine receptors A2B and A2A signaling. Deletion of A2B during CDI in a pre-clinical model in mice showed to be protective, for both intestinal damage and disease severity, while deletion of A2A demonstrated to promote worsened CDI (9).

So far, four isoforms of G-protein coupled adenosine receptors were described: A1, A2A, A2B, and A3, which are activated by adenosine, an endogenous, purine-based nucleoside that can be released by all eukaryotic cells (10). A1 and A3 belong to the Gi and G0 family of G-protein coupled receptors (GPCRs), while A2A and A2B are the Gs-coupled receptors. Their activation regulates the activity of adenylyl cyclase by increasing (A2A and A2B) or decreasing (A1 and A3) the synthesis of cAMP (11). Enteric glial cells (EGCs) are one of the intestinal cell populations that can express these receptors (12). Together with enteric neurons, EGCs forms the enteric nervous system (ENS) and are located on the mucosal, submucosal, and myenteric plexus, contributing to the homeostasis of the gut by regulating intestinal secretion, motility, and the epithelial barrier (13, 14). Under inflammatory conditions, including CDI, EGCs can also contribute to the inflammatory response by releasing S100B, a pro-inflammatory mediator and marker of reactive gliosis, when is released in high levels to the extracellular environment (5, 15).

Given that adenosine receptors have been shown to play an important role in CDI, we investigated how the modulation of adenosine receptors could contribute to the deleterious effects of *C. difficile* toxins on EGCs. Our findings showed that EGCs expressed all four subtypes of adenosine receptors, which differentially modulate IL-6 and S100B expression and cell death induced by TcdA and TcdB. These findings reveal new insight to understanding the role of these receptors during CDI and aid in the development of therapies able to decrease inflammation during infection, as well as decrease the damage to ENS components that can contribute to future intestinal dysfunction associated with post-CDI sequelae.

## Materials and methods

### Rat enteric glial cell culture and treatment

For our mechanistic study, we carried out the *in vitro* assays using an immortalized rat enteric glial cell (EGC) line PK060399egfr (ATCC CRL-2690, VA, United States). This cell exhibits similar morphology and functional properties to primary enteric glial cells from humans and mice (16). In addition, these cells expresses GFAP and S100B, which are enteric glial factors, and has been applied in a variety of mechanistic studies (5, 12, 17–22).

EGCs (PK060399egfr) were cultured in Dulbecco’s Modified Eagle’s Medium (DMEM, Gibco) and supplemented with 10% fetal bovine serum, 1% antibiotics (100 μg/mL penicillin and 100 μg/mL streptomycin, Gibco) and 1 mM sodium pyruvate (Gibco) at 37°C in a humidified incubator under 5% CO2 for no more than 20 passages. For all experiments, EGCs were released using 0.05% trypsin-EDTA for 5 min. Cells were incubated with 300 µM ATL313 (an A2a-selective adenosine receptor agonist), 3 µM SCH58261 (an A2a-selective adenosine receptor antagonist), 1 µM BAY60-6583 (an A2b-selective adenosine receptor agonist), 100 µM ATL801 (an A2b-selective adenosine receptor antagonist), 30 µM CI-IB-MECA (an A3AR-selective adenosine receptor agonist, 1066, Tocris), 0.01 µM MRS1220 (an A3AR-selective adenosine receptor antagonist, 1217, Tocris), 0.5 µM 666-15 (CREB inhibitor, 5661, Tocris), and 10 µM H89 dihydrochloride (PKA inhibitor, 2910, Tocris) added 1 hour prior to incubation with TcdA (50 ng/mL) or TcdB (1 ng/mL). In some experiments, EGCs were incubated with recombinant IL-6 (R&D system, 506-RL, 10 ng/mL and 100 ng/mL). All drug concentrations used were based on MTT assay results (**Supplementary Figure 1**). Purified TcdA and TcdB, produced by *C. difficile* strain VPI10463, were obtained from TechLab (VA, United States).

### Quantitative real-time PCR

EGCs (6x10^5^ cells/well) were seeded in 6-well plates and treated with TcdA or TcdB and pharmacologic modulators (ATL313, ATL801). After incubation, total RNA was extracted with a RNeasy Plus Mini Kit (Qiagen, Hilden, Germany) using QIAcube (Qiagen). RNA was quantified with a Qubit 3.0 fluorometer (Life Technologies) using a Qubit RNA BR Assay Kit (Invitrogen, Q10211). After DNA contamination was removed by RNA treatment with Dnase I (Invitrogen, 18068-015), a total of 1000ng of RNA was then reverse transcribed using an iScript cDNA Synthesis Kit (Bio-Rad, 1708891) according to the manufacturer’s protocol.

qPCR amplification of *A1*, *A2a*, *A2b*, *A3, S100B*, and *IL-6* in cell samples was performed in a CFX Connect system (Bio-Rad) with the following conditions: 95°C for 30 s, 40 cycles of 95°C for 5 s and 60°C for 30 s, and melt curve analysis from 60-95°C in 0.5°C increments for 2 s each. All PCRs were performed with iTaq Universal SYBR Green Supermix (Bio-Rad, 172-5124). The primer sets are listed in **Supplementary Table 1**.

### Immunofluorescence

EGCs (4x10^4^ cells/well) plated on 4-chamber glass tissue culture slides in a polystyrene vessel and treated with TcdA or TcdB for 18 h were fixed in 4% PFA solution (Alfa Aesar) in PBS for 30 min at room temperature and permeabilized with 0.5% Triton X-100 (Sigma-Aldrich) and 3% bovine serum albumin (BSA, Sigma) in PBS for 10 min at 4°C. After blocking with 5% normal donkey serum (Jackson ImmunoResearch, 017-000-121) in PBS for 30 min at room temperature, the cells were incubated with anti-A2A (Invitrogen, PA1-042, 1:50), anti-A2B (Invitrogen, PA5-72850, 1:100), anti-A3 (Invitrogen, PA5-36350, 1:50) or anti-IL-6 (1:20, R&D system, AF506) overnight at 4°C. After three washes with washing buffer (0.01% Tween 20 in PBS), the cells were incubated for 2 h with Alexa fluor 594-conjugated donkey anti-rabbit (Abcam, ab150064, 1:400), Alexa fluor 488-conjugated donkey anti-rabbit (Abcam, ab150073, 1:400) or Alexa fluor 488-conjugated donkey anti-goat (Abcam, ab150129, 1:400) antibodies, washed with PBS and mounted with ProLong Gold antifade reagent containing DAPI (Thermo Scientific, P36931). The samples were visualized by fluorescence microscopy (Evos, Thermo Scientific). The intensity of fluorescence was measured using Image J software.

### Cyclic AMP measurement

To measure the levels of cyclic adenosine monophosphate (cAMP) in EGCs treated with TcdA or TcdB for 18 h, the cAMP-Glo assay (Promega, V1501) was performed following the manufacturer’s instructions. The assay is based on the principle that cAMP stimulates protein kinase A (PKA) holoenzyme activity, decreasing available ATP and leading to decreased light production in a coupled luciferase reaction. Briefly, EGCs (10^4^ cells/well) were seeded in white tissue culture-treated 96-well plates (Falcon, solid white bottom) and treated with TcdA or TcdB for 18 h. Then, after removal of the supernatant, cells were incubated with 20 µL of cAMP-Glo lysis buffer with shaking at room temperature for 20 min. After this, 40 µL of cAMP-Glo detection solution (2.5 µL of PKA in 1000 µL cAMP-Glo reaction buffer) were added to each well and the plates were shaken for 60s and incubated at room temperature for 20 min. Then, 80 µL of kinase-Glo reagent were added to each well, and the plates were shaken for 60 s and incubated at room temperature for 10 min. The luminescence was recorded using a multi-mode reader (Synergy/HTX, Biotek), and the intrinsic reagent luminescence (no-cell, no-compound background control) was subtracted from the luminescence signals in the sample to obtain the relative luminescent units (RLUs).

### MTT assay

To determine the viability of the EGCs, cells (5x10^3^ cells/well) were seeded in 96-well plates and treated with several concentrations of adenosine receptors modulators (ATL313, SCH58261, BAY60-6583, ATL801, CI-IBMECA or MRS1220), inhibitors of CREB (666–15), and PKA (H89) for 18 h. Cells treated with DMSO (100%) was used as a positive control of cell death. Then, the cells were incubated with thiazolyl blue tetrazolium bromide (MTT, 0.5 mg/mL reconstituted in supplemented DMEM, Sigma-Aldrich, M2128) for 2 h at 37°C in a humidified incubator under 5% CO_2_. After removal of the MTT solution, 150 µL of 100% dimethyl sulfoxide was added to each well. The plates were then shaken for 2 min at room temperature, and the absorbance of the reaction at 570 nm was measured using an ELISA reader.

### Realtime-glo annexin V apoptosis assay

Cell death was evaluated with a live cell real-time assay (Realtime-Glo annexin V apoptosis assay, Promega, JA1000), following the manufacturer’s instructions. EGCs (10^4^ cells/well) were seeded in white tissue culture-treated 96-well plates (Falcon, solid white bottom) and treated with TcdA or TcdB for 18 h in the presence or absence of 300 µM ATL313 (an A2a-selective adenosine receptor agonist), 3 µM SCH58261 (an A2a-selective adenosine receptor antagonist), 1 µM BAY60-6583 (an A2b-selective adenosine receptor agonist), 100 µM ATL801 (an A2b-selective adenosine receptor antagonist), 30 µM CI-IBMECA (an A3AR-selective adenosine receptor agonist), 0.01 µM MRS1220 (an A3AR-selective adenosine receptor antagonist), 0.5 µM 666-15 (CREB inhibitor), and 10 µM H89 (PKA inhibitor) added one hour prior to *C. difficile* toxin challenge. Then, 200 µL of 2x detection reagent (2 µL of annexin NanoBit substrate, 2 µL of CaCl_2_, 2 µL of annexin V-SmBit and 2 µL of annexin V-LgBit in 1000 µL of prewarmed supplemented DMEM) were added to each well, and the cells were incubated at 37°C in a humidified incubator under 5% CO_2_. The luminescence was recorded using a multi-mode reader (Synergy/HTX, Biotek), and the intrinsic reagent luminescence (no-cell, no-compound background control) was subtracted from the luminescence signals in the sample, then divided by the mean of control cells to obtain the relative luminescent units (RLUs).

### Caspase 3/7 activity assay

Caspase 3/7 activity was evaluated with a Caspase-Glo assay kit (Promega, G8091) following the manufacturer’s instructions. EGCs (10^4^ cells/well) were seeded in white tissue culture-treated 96-well plates (Falcon, solid white bottom) and treated with TcdA or TcdB for 18 h in the presence or absence 300 µM ATL313 (an A2a-selective adenosine receptor agonist), 100 µM ATL801 (an A2b-selective adenosine receptor antagonist) or 30 µM CI-IBMECA (an A3AR-selective adenosine receptor agonist) added one hour prior to *C. difficile* toxin challenge. Then, the plates containing the cells were removed from the incubator for 15 min. A volume of 100 µL of Caspase-Glo reagent was added to each well, and the wells were mixed with a plate shaker at 500 rpm for 30 s. The plates were incubated for 2 h at room temperature. Then, the luminescence of each sample was acquired in a multi-mode reader (Synergy/HTX, Biotek) to obtain the relative luminescent units (RLUs).

### Knockdown of A2A and A2B in EGCs

To ensure successful knockdown of the respective genes, we performed a lentiviral-mediated CRISPR/Cas9 genome editing using three different targets for each one: *A2A* sgRNA CRISPR/Cas9 (target 1-6: 5’ CTCCTCGGTGTACATCACGG 3’, target 2-102: 5’ AGAAGTTGGTGACGTTCTGC 3’, target 3-297: 5’ GAATTCGGATGGCGATGTAG 3’) and *A2B* sgRNA CRISPR/Cas9 (target 1-12: 5’ GACGCAGGACGCGCTGTACG 3’, target 2-288: 5’ GGTCGACAGCCACCGCCAAG 3’, target 3-320: 5’ TCACCTGAGCGGGACGCGAA 3’). Plasmids were transformed into NEB^®^ Stable Competent *Escherichia coli* (High Efficiency) following the manufacturer’s protocol. Clones were confirmed by Sanger sequencing.

In order to produce lentiviral particles for viral transduction, integration and consequently constitutive expression, we packaged our lentiviral sgRNAs seeding a total of 3.5x10^6^ HEK 293T/17 cells in 10 cm dishes. These cells were maintained with Iscove’s modified Dulbecco’s medium (IMDM) supplemented with 10% bovine calf serum (BCS) and 0.1% of gentamicin. Twenty-four hours later, cell medium was changed to serum-free IMDM. One hour later, cells were co-transfected with 15 ug of each different construct, 12.84 ug of psPAX2, and 2.58 ug of pMD2.G (VSV-g) using a 1:3 ratio of 1 mg/mL of polyethylenimine (PEI) pH 7.0. Seventy-two hours after transfection, cell culture supernatants were collected, centrifuged to remove cell debris, aliquoted, and frozen at -80° C.

To establish an EGC line PK060399egfr knockdown of A2A and A2B, cells were infected with lentiviral particles containing the plasmids for the target genes overnight. The medium was removed and a supplemented DMEM containing puromycin (0.2 µg/mL) was added and changed every 48h for 7 days. The knockdown of A2A and A2B was confirmed by immunofluorescence (**Supplementary Figure 2**).

### Western blot analysis

To evaluate the protein levels of pCREB and PKA, EGC lines (6×10^5^ cells/well) were seeded in six-well plates and treated with TcdA or TcdB for 1.5, 3.5, and 4h. After incubation, the supernatant was removed, the cells were lysed using RIPA lysis buffer (Thermo Fisher Scientific, containing EDTA and phosphatase-free protease inhibitor) and centrifuged (17 min, 4°C, 13000 rpm), and the supernatant was collected. Protein concentrations were determined through the bicinchoninic acid assay according to the manufacturer’s protocol (Thermo Fisher Scientific). Reduced 40 µg protein samples (prepared with sample reducing agent- Invitrogen- and protein loading buffer-LI-COR) were denatured at 99°C for 5 min, separated on NuPAGE 4-12% BIS-Tris gel (Invitrogen), and transferred to nitrocellulose membranes (Life Technologies) for 2 h. The membranes were then immersed in iBind fluorescent detection solution (Life technologies) and placed in a iBIND automated Western device (Life Technologies) overnight at 4°C for blocking, incubating with primary antibodies (mouse anti-α-tubulin, 1:2000, Sigma-Aldrich, T6199; rabbit pCREB, 1:500, Novus biological, NB300-273; rabbit phosphor-PKA, 1:500, Invitrogen, PA5-64489) and secondary antibodies (Cy3-conjugated AffiniPure donkey anti-rabbit, 711-165-152, 1:1000, Jackson Immuno-Research and Cy5-conjugated AffiniPure donkey anti-mouse, 715-175-150, 1:1000, Jackson ImmunoResearch). Then, the membranes were immersed in ultrapure water, and fluorescent signal was detected using the Typhoon system (GE healthcare). Densitometric quantification of bands was performed using ImageJ software (NIH, Bethesda, MD, USA).

### ELISA assay for pCREB measurement

Protein lysates were extracted from seeded EGC lines (6×10^5^ cells/well), which were plated in 6-well plates and treated with TcdA or TcdB for 4 h, using radioimmunoprecipitation assay (RIPA) buffer (20 mM Tris, 150 mM NaCl, 1% Nonidet P-40, 0.5% sodium deoxycholate, 1 mM EDTA, 0.1% SDS, adjusted to pH 7.5) containing protease inhibitor cocktail (Sigma-Aldrich) and phosphatase inhibitors (Sigma-Aldrich). Lysates were centrifuged at 13000 rpm for 15 min, and the supernatant was used to perform the protein assay using the bicinchoninic acid assay (Thermo Fisher Scientific). The transcription factor pCREB was measured using a commercial ELISA kit (R&D Systems, DYC2510-2) according to the manufacturer’s instructions. The absorbance (450 nm) was determined using an Epoch plate reader (BioTek). Levels of these cytokines were measured as picograms per milligram of protein.

### Mice

Male C57BL/6 mice WT, A2A, and A2B Knockout (Jackson Laboratory, Farmington, US, 8 weeks of age) were housed in temperature-controlled rooms under 12 h light-dark cycles. The animals received water and food *ad libitum*. All surgical procedures and treatments performed with C57BL/6 mice were conducted in accordance with the Guidelines for Institutional and Animal Care and Use of the University of Virginia, Charlottesville, US. The protocol has been approved by the committee on the Ethics of Animal Experiments of the University of Virginia (Protocol number: 4096).

### *C. difficile* infection model

Our murine CDI model was established as previously described (5, 23, 24). To disrupt the microbiota and facilitate *C. difficile* colonization or infection, wild type (WT) or Knockout (*A2A* or *A2B*KO) C57BL/6 mice (n=8-9 for each group) received an antibiotic treatment (0.035 mg per mL gentamicin, 850 U per mL colistin, 0.215 mg per mL metronidazole, and 0.045 mg per mL vancomycin) in drinking water for 3 days. After 1 day off antibiotics, an intraperitoneal injection of clindamycin (32 mg per kg) was given 1 day before *C. difficile* challenge. Then, 10^5^ CFU of the vegetative *C. difficile* strain VPI10463 (ATCC 43255, *tcdA+tcdB+cdtB-*) was administered by oral gavage. Control mice received the same vehicle. Mouse weights and the development of disease symptoms were monitored daily. Animals that became moribund or lost >20% of their body weight were euthanized.

As shown in our previous study, uninfected *A2A*KO (25) or *A2B*KO (26) mice did not exhibit intestinal damage/infiltration of inflammatory cells (**Supplementary Figure 3** and **Supplementary Figure 4**) or changes on synthesis of inflammatory mediators.

### Measurement of microscopic damage

Mice cecum tissues from day 3 p.i. were fixed in 10% neutral buffered formalin for 20h, dehydrated and embedded in paraffin. Cecal sections (5 µm) were then stained with hematoxylin and eosin staining (H&E) and examined using light microscopy. Histopathological scores were performed by a blinded investigator, using a previously described method (5, 27). Histopathological scores were determined by quantifying the intensity of epithelial tissue damage (0-3, 0-no damage, 1-mild, 2-moderate, 3-extensive), edema in mucosa/submucosa layer (0-3) and cell infiltration (0-3). The total histological damage score was measured by the sum of the three parameters evaluated.

### Immunohistochemistry

The immunostaining for A2A, A2B, A3, HuC/D and IL-6 in cecum samples was performed at the UVA Biorepository and Tissue Research Facility. Briefly, Sections (4 µm thick) were prepared from paraffin-embedded mouse cecum tissues. After deparaffinization, antigens were recovered by incubating the slides in citrate buffer (pH 6.0) for 20 min at 95°C. Endogenous peroxidase was blocked with 3% H_2_O_2_ for 10 min to reduce nonspecific binding. The sections were then incubated with the primary antibodies A2A (PA1-042, Invitrogen, 1:200), A2B (Invitrogen, PA5-72850, 1:400), A3 (Invitrogen, PA5-36350, 1:100), HuC/D (Abcam, ab184267, 1:100), or IL-6 (Bioss, BS-0379R, 1:100), overnight. The sections were then incubated for 30 min with polymer (K4061, Dako). The antibody binding sites were visualized by incubating the samples with diaminobenzidine–H_2_O_2_ (DAB, Dako) solution. Sections incubated with antibody diluent without a primary antibody were used as negative controls. Antibody specificity was evaluated using positive controls for each antibody in the mouse large intestine (data not shown).

The amounts of DAB products after immunostaining were estimated from at least 6-8 and from different areas of each section (from 8-9 specimens per group) for mouse samples, at 200X magnification using Image J software.

### Terminal deoxynucleotidyl transferase-mediated deoxyuridine triphosphate nick-end labeling assay

EGC death in cecum tissues of infected WT, A2AKO, and A2BKO mice were investigated using a TdT-mediated dUTP nick end-labeling (TUNEL) assay and S100B immunoassaying (a marker for EGCs) according to the manufacturer’s protocol (Click-iT Plus TUNEL assay, C10618, Invitrogen). After TUNEL staining was performed according to the manufacturer’s protocol, tissues were blocked using 5% donkey serum in PBS for 30 min at room temperature followed by incubation with S100B primary antibody (1:200, NBP-53188, Novus Biologicals) overnight at 4°C. Then, the cells were washed twice in PBS solution with 0.1% Tween 20 and incubated with secondary antibody (1:400, ab150073, Abcam) for 2 h at room temperature. After this time, cells were washed, stained with 4’,6-diamidino-2-phenylindole, dihydrochloride (DAPI, Invitrogen, D1306), washed in PBS, and mounted with antifade mounting medium (Vector). Cells were acquired using Leica DFC900 GTC VSC-11976 system (Leica) of at least three-eight distinct fields of each well per group from 2-3 different experiments. Cells were counted using ImageJ software (NIH, Bethesda, MD, USA). Fluorescence intensity of S100B was also measured by using ImageJ software (NIH, Bethesda, MD, USA).

### Statistical analysis

The data are presented as the mean ± standard error of the mean (SEM). Student’s t test or one- or two-way analysis of variance (ANOVA) followed by the Turkey test was used to compare means. *P*< 0.05 was considered to indicate significance.

## Results

### *C. difficile* toxins upregulate A2A and A3 and downregulate A2B in EGCs

**Figure 1:**
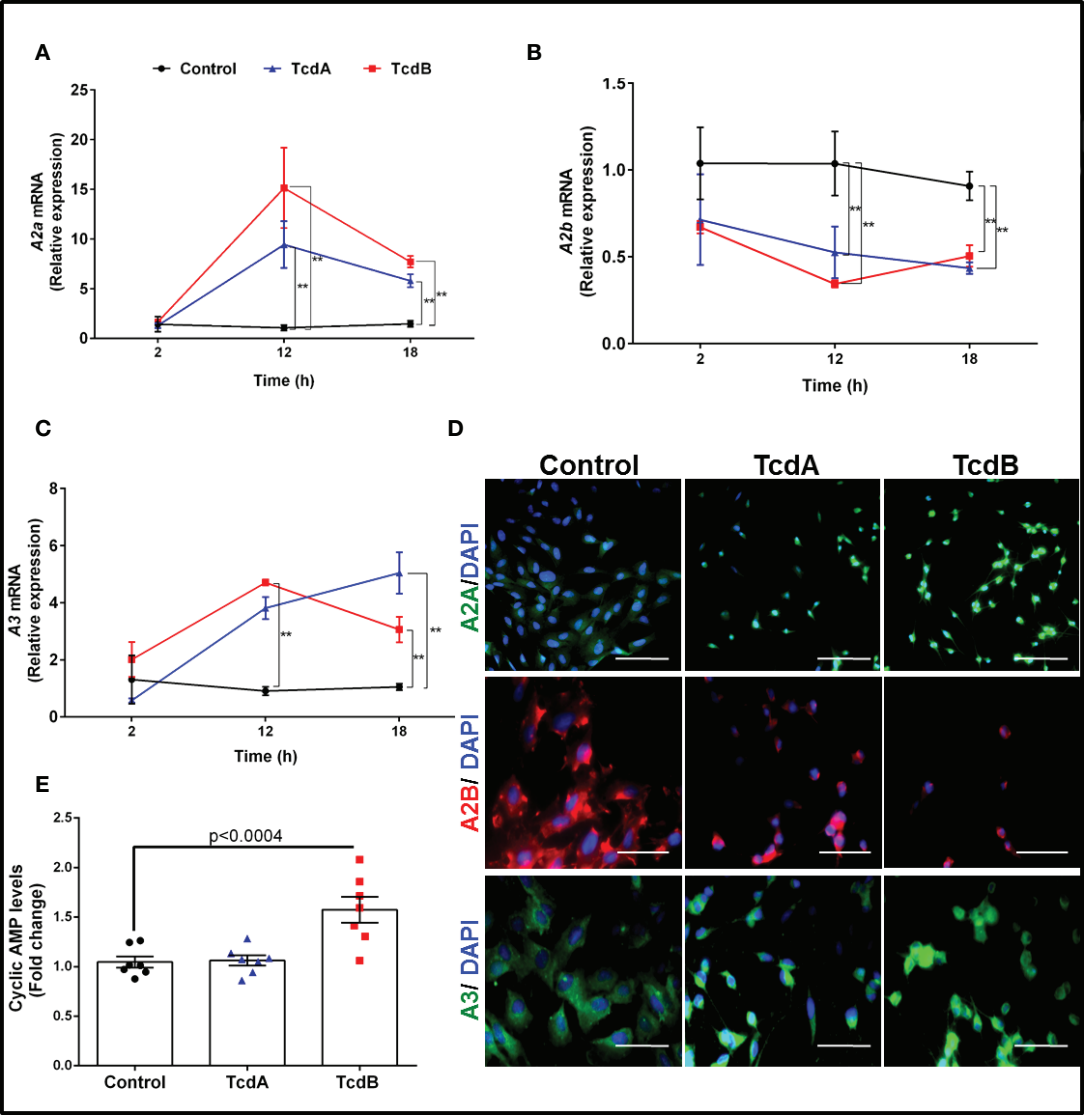
TcdA and TcdB upregulate expression of anti-inflammatory adenosine receptors (A2A and A3) and downregulate the proinflammatory adenosine receptor (A2B) in enteric glia *in vitro*. **(A)** Levels of *A2A*, **(B)** *A2B* and **(C)** *A3* gene expression (mean ± s.e.m) by qPCR in EGC/PK060399 challenged with TcdA (50 ng/mL) and TcdB (1 ng/mL) (n=6). Two-way ANOVA followed by Turkey test was used. **(A–C)** **p<0.0001. **(D)** Representative photomicrographs of *A2A*, *A2B* and *A3* immunostaining, and DAPI (blue) as a nuclear staining in EGC exposed to TcdA and TcdB at 18 h incubation. Scale bars, 100 µm (A2A photomicrographs) and 50 µm (A2B and A3 photomicrographs). **(E)** cyclic AMP levels (mean ± s.e.m) evaluated by a luminescence assay in EGCs challenged with TcdA and TcdB at 18h incubation (n=6). One-way ANOVA followed by Turkey test was used. p value is indicated in the graph. These experiments are from three different replicates.

To identify the expression profile of adenosine receptors in EGCs challenged by TcdA and TcdB, we performed a qPCR to A1, A2A, A2B, and A3. We found that TcdA and TcdB upregulated A2A and A3 and downregulated A2B at 12 h incubation (p<0.0001, **Figures 1A–C**), which persisted at 18 h incubation (p<0.0001, **Figure 1A**), as also shown in our immunofluorescence images (**Figure 1D**). Transcripts of A1 were also found to be expressed by EGCs but were not changed by challenge with either toxin (**Supplementary Figure 5**).

Given that activation of adenosine receptors leads to increase or decrease cAMP production, we evaluated the levels of this molecule in the EGCs challenged by *C. difficile* toxins. We found that TcdB, but not TcdA, increased the levels of cAMP compared to the control cells (p<0.0004, **Figure 1E**).

To investigate the tissue distribution of A2A, A2B and A3 during CDI, we performed an immunohistochemistry assay. We found that the percentage of positive immunostaining for all these three adenosine receptors are increased during CDI compared to uninfected mice (**Supplementary Figures 6A, B**). As shown in **Supplementary Figure 6B**, although all three receptors can be found in ENS components, such as in myenteric plexus, although A3 is not as expressed in the cecum samples.

### EGC death induced by TcdA and TcdB are differentially modulated by adenosine receptors

**Figure 2:**
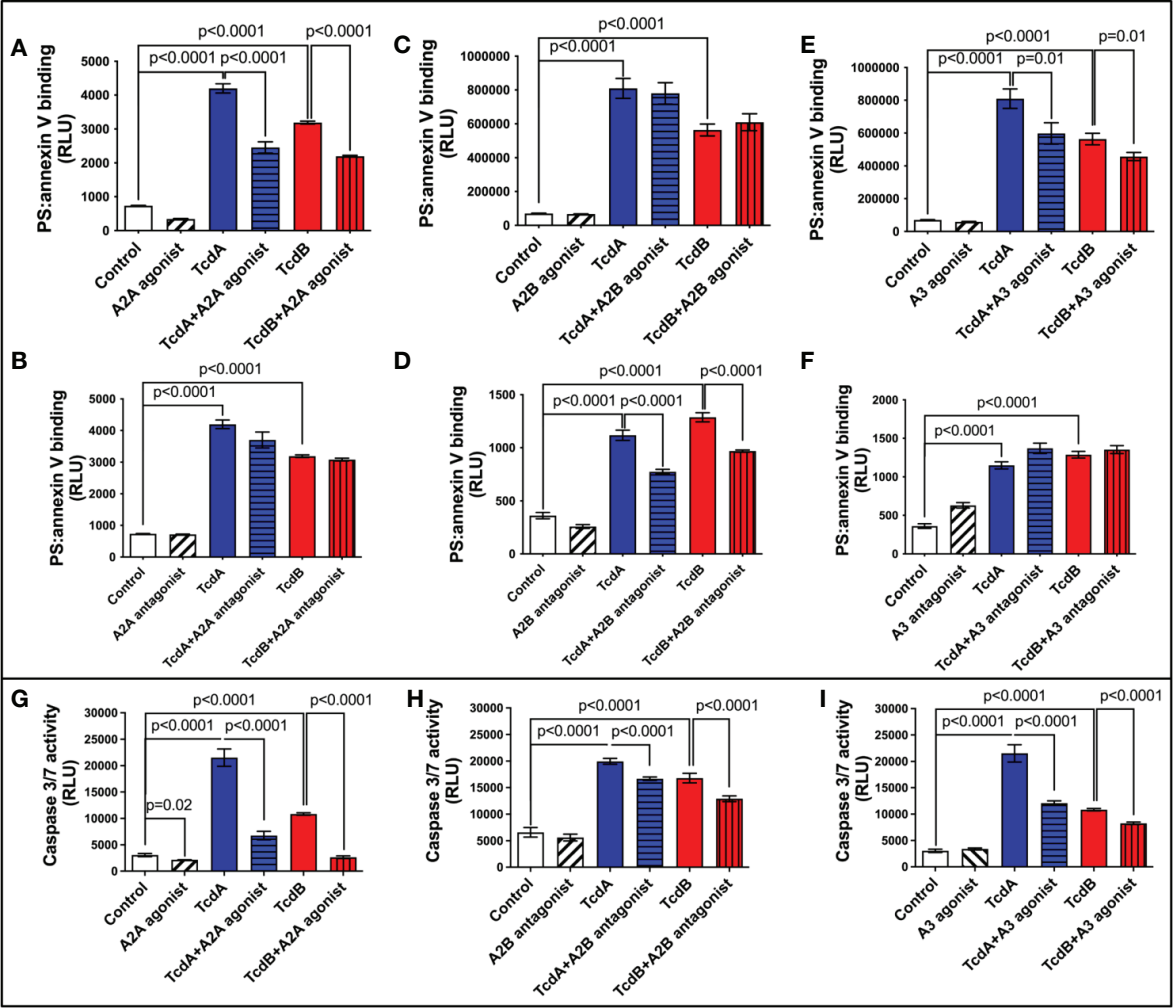
Modulation of adenosine receptors decrease EGCs death induced by *C. difficile* toxins. **(A–F)** Cell death analyzed by RealTime-Glo annexin V apoptosis assay (mean ± s.e.m, n=6) in enteroglial cell (EGC/PK060399) challenged with TcdA and TcdB for 18 h in the presence or absence of **(A)** 300 µM ATL313 (an A2a-selective adenosine receptor agonist), **(B)** 3 µM SCH58261 (an A2a-selective adenosine receptor antagonist), **(C)** 1 µM BAY60-6583 (an A2b-selective adenosine receptor agonist), **(D)** 100 µM ATL801 (an A2b-selective adenosine receptor antagonist), **(E)** 30 µM CI-IBMECA (an A3AR-selective adenosine receptor agonist), and **(F)** 0.01 µM MRS1220 (an A3AR-selective adenosine receptor antagonist) added one hour prior to *C*. *difficile* toxin challenge. **(G–I)** Activity of caspase 3/7 analyzed by luminescence assay (mean ± s.e.m, n=6) in enteroglial cell (EGC/PK060399) challenged with TcdA and TcdB for 18 h in the presence or absence of **(G)** 300 µM ATL313, **(H)** 100 µM ATL801 and **(I)** 30 µM CI-IBMECA added one hour prior to *C*. *difficile* toxin challenge. **(A–I)** One-way ANOVA followed by Turkey test was used. These experiments are from two different replicates.

TcdB has been shown to induce EGCs death (18–20), which can contribute to intestinal dysfunction post-infection. We assessed whether adenosine receptors are involved in cell death induced by *C. difficile* toxins by evaluating the binding of annexin and phosphatidylserine. We found that A2A agonist (ATL313), but not A2A antagonist (SCH58261), decreased the levels of annexin and phosphatidylserine binding induced by TcdA and TcdB in EGCs (p<0.0001, **Figures 2A, B**). Blockage of A2B (ATL801), but not activation of A2B (Bay60-6583), reduced the EGC death induced by both toxins (p<0.0001, **Figures 2C, D**). A3 agonist (CI-IBMECA), but not A3 antagonist (MRS1220), diminished EGC death induced by both toxins (p=0.01, **Figures 2E, F**).

Next, we evaluated the activity of caspase 3/7, as a marker of apoptosis, in EGCs challenged with *C. difficile* toxins and pretreated with A2A agonist (ATL313), A2B antagonist (ATL801), and A3 agonist (CI-IBMECA). We found that A2A agonist (ATL313), A2B antagonist (ATL801), and A3 agonist (CI-IBMECA) decreased the levels of caspase 3/7 induced by both toxins (p<0.0001, **Figures 2G–I**).

### *A2B*, but not *A2A*, deletion prevents EGC death *in vitro* and intestinal cell death and enteric neuron loss during CDI in mice

**Figure 3:**
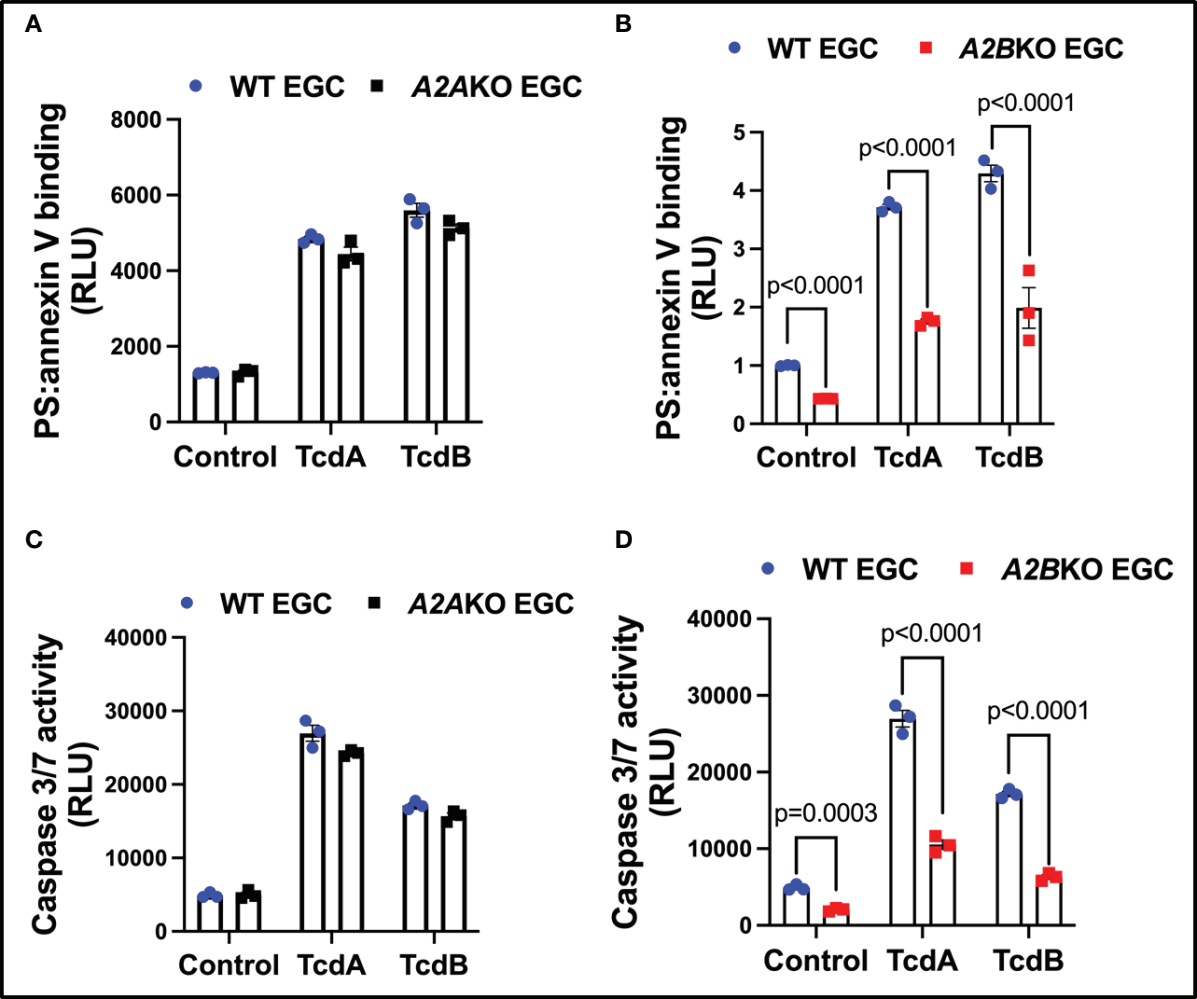
Knockdown of *A2B*, but not *A2A*, attenuates EGCs death induced by *C. difficile* toxins. **(A, B)** Cell death analyzed by RealTime-Glo annexin V apoptosis assay (mean ± s.e.m, n=6) in WT, *A2AKO* and *A2BKO* enteroglial cell (EGC/PK060399) challenged with TcdA and TcdB for 18 h. **(C, D)** Activity of caspase 3/7 analyzed by luminescence assay (mean ± s.e.m, n=6) in WT, *A2AKO* and *A2BKO* enteroglial cell (EGC/PK060399) challenged with TcdA and TcdB for 18 h. **(A–D)** One-way ANOVA followed by Turkey test was used. These experiments are from two different replicates.

To confirm that A2A and A2B determine EGCs death, we knockdown these receptors in EGCs to determine their role in caspases activity and cell death. Knockdown of A2B, but not A2A, decreased EGCs death (p<0.0001, **Figures 3A, B**) and activity of caspases 3/7 (p<0.0001, **Figures 3C, D**) in EGCs challenged with TcdA and TcdB *in vitro*.

**Figure 4:**
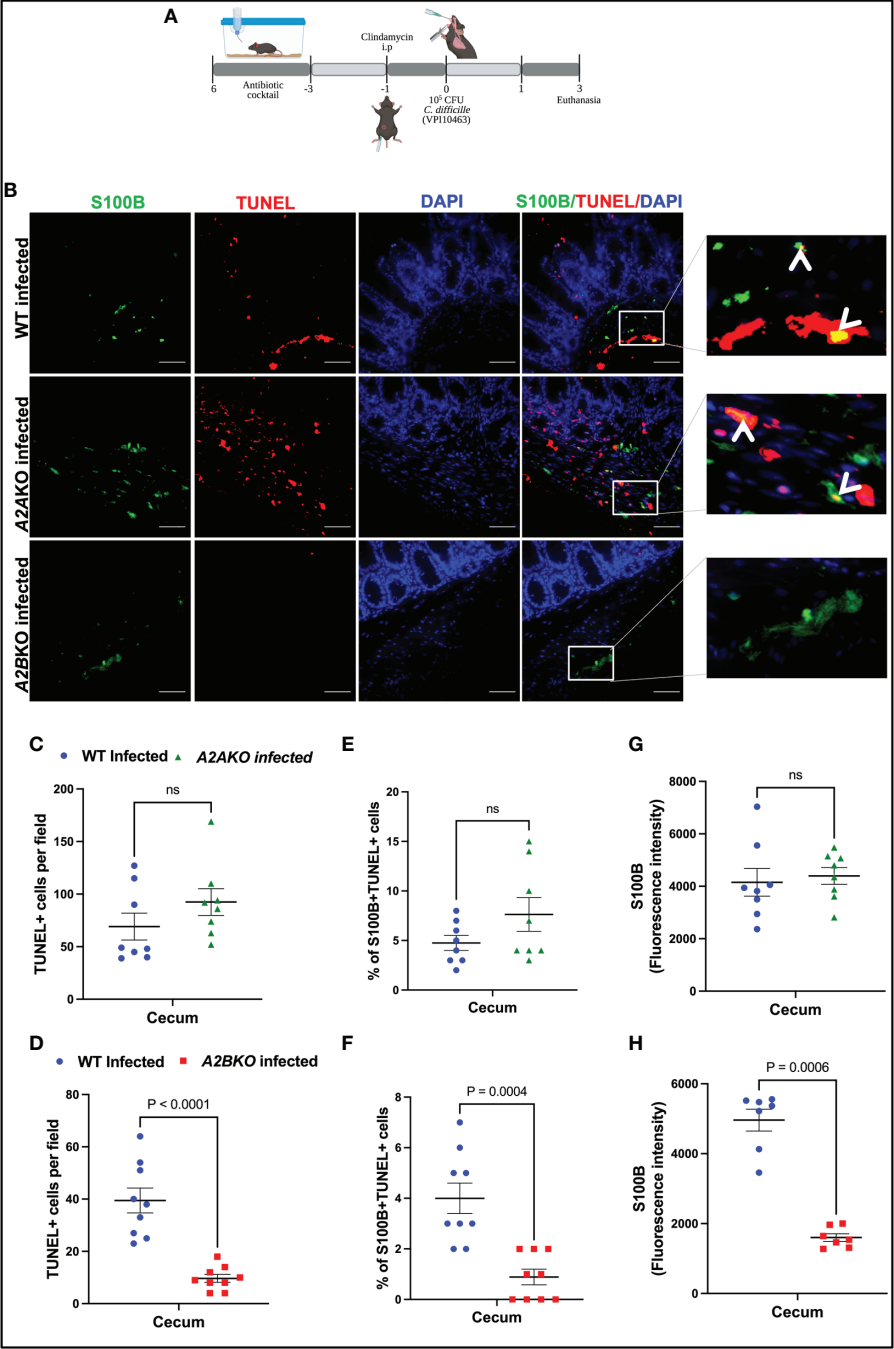
Deletion of *A2B*, but not *A2A*, prevents EGCs death induced by *C*. *difficile* infection. **(A)** Scheme of *C*. *difficile* infection (CDI) in mice. **(B)** Representative photomicrographs of S100B (green) immunostaining, TUNEL (red), and DAPI (blue) nuclear staining in cecum samples from WT, *A2*AKO and *A2*BKO mice on day 3 post *C*. *difficile* infection. **(C, D)** Analysis of TUNEL+ cells (marker of dead cells) in cecum samples from WT, *A2*AKO and *A2*BKO mice on day 3 post *C*. *difficile* infection (n=8-9). **(E, F)** Analysis of S100B+Tunel+ cells (dead EGCs) in cecum samples from WT, *A2*AKO and *A2*BKO mice on day 3 post *C*. *difficile* infection (n=8-9). **(G, H)** Fluorescence intensity of S100B immunostaining measured by ImageJ software in cecum samples from WT, *A2*AKO and *A2*BKO mice on day 3 post *C*. *difficile* infection (n=8-9). **(C–H)** Mann-Whitney’s test was used. ns, not significant.

Next, using *A2A* and *A2B* knockout mice, we investigated the role of these receptors on intestinal cell death, including EGC death during CDI in mice (**Figure 4A**). *A2B* knockout infected mice exhibited less epithelial damage (p=0.02), cell infiltrate (p<0.0001) and edema (p<0.0001) compared to WT infected mice (**Supplementary Figures 3A, B**). Whereas *A2A* knockout infected mice showed higher cell infiltrate (p<0.0001) and edema (p=0.002) score compared to WT infected mice (**Supplementary Figures 4A, B**).

A2B deletion, but not A2A, decreased the intestinal cell death in cecum of infected mice compared to WT mice (p<0.0001, **Figures 4B–D**). Among these cells, using a marker for EGCs, S100B, we identified that EGCs are one of the cells dying during CDI (**Figure 4B**). As shown in **Figure 4E**, *A2A* deletion, again, did not prevent the increase of the percentage of S100B+TUNEL+ cells induced by CDI in mice (**Figures 4B, E**). However, *A2B* deletion decreased the EGC death in infected mice compared to the WT mice (p=0.0004, **Figures 4B, F**). A2B deletion decreased S100B levels in infected mice (p=0.0006, **Figures 4G, H**).

Another important component of the ENS are the enteric neurons. We performed an immunohistochemistry for HuC/D, a specific enteric neuronal marker in the gut, to detect the impact of deletion of adenosine receptors for the loss of neurons during CDI. As shown in **Supplementary Figure 7**, infected A2BKO mice, but not infected A2AKO mice, exhibited higher positive HuC/D immunostaining than WT infected mice (p=0.002, **Supplementary Figures 7A–B**), suggesting that A2B may be involved in loss of enteric neurons during CDI.

### Inhibition of PKA or CREB reduces EGC death induced by TcdA and TcdB

**Figure 5:**
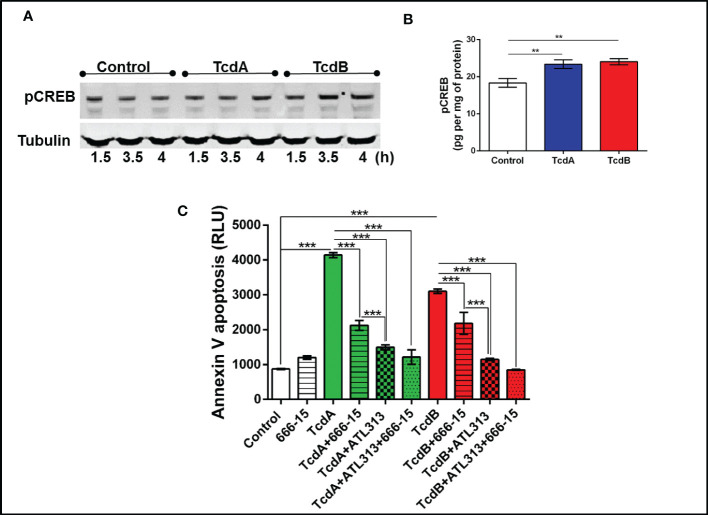
C. *difficile* toxins increases the levels of phosphorylated CREB, which are involved in EGCs death induced by these toxins *in vitro*. **(A)** Representative Western Blot images showing phosphorylated CREB and tubulin (loading control) protein expression. **(B)** levels of pCREB (mean ± s.e.m.) measured by ELISA in EGCs challenged with TcdA and TcdB for 4h. **(C)** Cell death analyzed by RealTime-Glo annexin V apoptosis assay (mean ± s.e.m, n=6) in enteroglial cell (EGC/PK060399) challenged with TcdA and TcdB for 18 h in the presence and/or absence of 0.5 µM 666-15 (a CREB inhibitor) and 300 µM ATL313 (an A2a-selective adenosine receptor agonist), added one hour prior to *C*. *difficile* toxin challenge. **(B, C)** One-way ANOVA followed by Turkey test was used. These experiments are from two different replicates. ***p<0.0001, **p<0.001.

To better understanding the mechanism involved on antiapoptotic effect mediated by A2A activation in EGC challenged with *C. difficile* toxins, we investigated whether PKA-CREB signaling could be involved in this process. We found that phosphorylated PKA (p=0.03, **Supplementary Figures 8A, B**) increased at 3.5h post-*C. difficile* toxins challenge and pCREB (p<0.001, **Figures 5A, B**, **Supplementary Figure 9**) increased in EGCs challenged with C. *difficile* toxins at 4h incubation time point. Inhibition of PKA (p<0.0001, **Supplementary Figure 8C**) and CREB (p<0.0001, **Figure 5C**) decreased the levels of phosphatidylserine binding to annexin induced by TcdA and TcdB in EGCs. In addition, simultaneous activation of A2A and inhibition of CREB did not function better than A2A agonist alone in preventing EGC death induced by TcdA and TcdB, indicating that another pathway plays a role in the anti-apoptotic effects generated by the activation of A2A (p<0.0001, **Figure 5C**).

### TcdA- and TcdB-induced *IL-6* expression in EGCs is decreased by A2A agonist and increased by A2B antagonist

**Figure 6:**
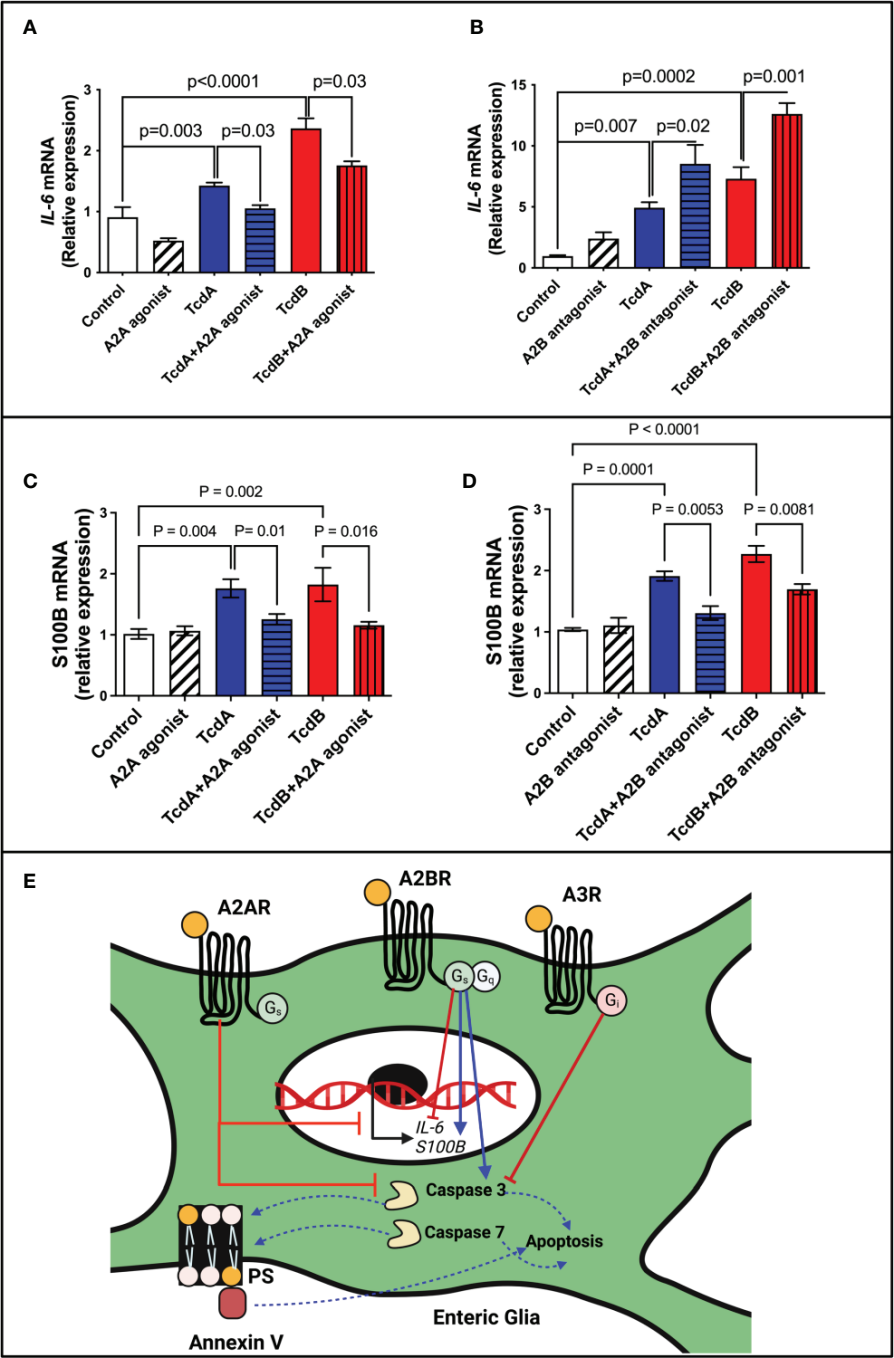
Regulation of IL-6 and S100B expression by adenosine receptors in EGCs challenged with *C. difficile* toxins. **(A, B)** Analysis of *IL-6* gene expression (mean ± s.e.m) by qPCR in enteroglial cell (EGC/PK060399) challenged with TcdA and TcdB for 18h in the presence or absence of **(A)** 300 µM ATL313 (an A2a-selective adenosine receptor agonist), and **(B)** 100 µM ATL801 (an A2b-selective adenosine receptor antagonist). **(C, D)** Analysis of *S100B* gene expression (mean ± s.e.m) by qPCR in enteroglial cell (EGC/PK060399) challenged with TcdA and TcdB for 18h in the presence or absence of **(C)** 300 µM ATL313 (an A2a-selective adenosine receptor agonist), and **(D)** 100 µM ATL801 (an A2b-selective adenosine receptor antagonist).These experiments are from two different replicates. **(A–D)** One-way ANOVA followed by Turkey test was used. **(E)** Proposed model of the role of adenosine receptors on deleterious effects induced by *C*. *difficile* toxins on EGCs: *C*. *difficile* toxins (TcdA and TcdB) upregulate A2A and A3 and downregulate A2B receptors in EGCs. Activation of A2A and A3, as well as inhibition of A2B lead to inhibition of caspase 3/7 activation and consequently inhibits cell death induced by *C*. *difficile* toxins in a manner independently of PKA-CREB signaling. While A2A modulates S100B negatively, A2B promotes a positive effect in the expression of this mediator in EGCs challenge by *C*. *difficile* toxins. While the modulation of IL-6 expression by adenosine receptors in EGCs challenged by TcdA and TcdB seem to be complex, A2A decreasing its expression and A2B showing an opposite effect.

To evaluate whether *IL-6* may be involved in the modulation of apoptosis by adenosine receptors, A2A and A2B, we assessed *IL-6* mRNA expression and its protein levels induced by *C. difficile* toxins. A2A agonist (ATL313) decreased the *IL-6* expression induced by TcdA and TcdB in EGCs (p=0.03, **Figure 6A**; TcdA/p<0.0001, TcdB/p=0.0002, **Supplementary Figure 10**), whereas A2B antagonist (ATL801) had the opposite effect (TcdA/p=0.02, TcdB/p=0.001, **Figure 6B**; TcdB/p=0.0001, **Supplementary Figure 10**).

However, when antiapoptotic CREB was blocked, *IL-6* expression did not significantly change in toxin-challenged EGCs (**Supplementary Figure 11**). Further, IL-6 by itself did not promote EGCs death (**Supplementary Figure 12**) indicating that IL-6 may not directly be involved in adenosine receptor-mediated apoptotic effects.

Although A2B blockage increase IL-6 expression in EGCs challenged by *C. difficile* toxins *in vitro*, A2B deletion during CDI in mice decreased IL-6 levels (**Supplementary Figure 13**), suggesting that the interaction of EGCs with other cells *in vivo* can modify this phenotype seen *in vitro*.

Given that S100B has been associated to cell death in other inflammatory conditions (15, 28) and our *in vivo* data suggested that A2B was involved in increased S100B during CDI, we evaluated whether A2B and A2A were involved directly in the upregulation of S100B induced by TcdA and TcdB in EGCs. We found that A2A agonist or A2B antagonist decreased S100B upregulation induced by both toxins (**Figures 6C, D**).

## Discussion

We found that EGCs expressed all four adenosine receptors, and *C. difficile* toxins upregulated the adenosine anti-inflammatory-related receptors (A2A and A3) and downregulated the proinflammatory-related receptor (A2B). Increased A2A expression was also found in murine macrophages, bone marrow-derived macrophages challenged by lipopolysaccharides (LPS) (29, 30), and intestinal epithelial cells challenged with TcdA (26, 31). We have previously shown that *C. difficile* toxins promoted upregulation of A2B in human intestinal epithelial cell lines (26). Decreased A3 protein expression was reported in intestinal epithelial cells from mice with colitis induced by dextran sulfate sodium (DSS) (32).

In this study, we found that activation of anti-inflammatory A2A and A3 and blockage of pro-inflammatory A2B decreased EGC death induced by either TcdA or TcdB. However, it was only the knockdown or deletion of A2B that reduced EGC death, *in vitro* and in a murine model of CDI, respectively. These findings indicate the critical role of A2B in *C. difficile* toxin-induced EGC death. A2A deletion did not increase the EGCs death induced by *C. difficile* toxins which we speculate to be due to the presence of A3 receptors, whose activation promotes an antiapoptotic effect. As previously reported, deletion of *A2B* was protective against CDI, decreasing the intestinal epithelial damage and release of pro-inflammatory mediators *in vivo* (25, 26). Inhibition of A2A has been shown by others to induce epithelial cell death in a lung cancer model (33). A2B antagonist has been shown to decrease neuronal apoptosis induced by oxygen and glucose deprivation in hippocampus of rats (34). On the other hand, A3 agonist has been shown to be proapoptotic when used in high concentrations (35–37) and anti-apoptotic in low concentrations (38, 39). One of the anti-apoptotic effects of A3 agonist was demonstrated in retinal ganglion cells damaged by laser-induced ocular hypertension (39) and in myocardium injured by a reperfusion/reoxygenation model (38). One of the mechanisms by which adenosine receptors modulated a decrease in EGC death induced by *C. difficile* is *via* decreasing caspase 3/7 activation, as shown in our findings. Together with caspases 8, 9 and 10 (initiator caspases), caspases 3, 6 and 7 (effector caspases) are part of the apoptotic caspases. Caspases 3 and 7 are found in the cytoplasm of cells in an inactive homodimers form that became proteolytic active once the initiators and activated executioner caspases cleave the linker that separates their large and small catalytic subunits, culminating in apoptosis (40). Of note, *C. difficile* toxins promotes death of intestinal epithelial cells by activation of caspases 3 (2, 3, 41).

Activation of CREB in other cells, such as neuronal cells, has been protective, promoting antiapoptotic effect by stimulating expression of antiapoptotic mediators (42–44). Our data revealed that PKA/CREB signaling was increased by toxin challenge but was not required for the antiapoptotic effect of A2A activation in EGCs challenged with *C. difficile* toxins suggesting other pathways involved in the A2A mediated protection. More investigations are needed to better understand the intracellular signaling involved in this process.

We also found that stimulating A2A decreased *IL-6* upregulation induced by *C. difficile* toxins. Consistent with this finding, in murine bone marrow-derived macrophages and colitis induced by pathogenic CD45RB^high^ Th cells, loss of A2A receptor resulted in higher proinflammatory cytokine synthesis (30, 45). Deletion of A2A in murine bone marrow-derived macrophages led to increase of basal phosphorylation of p38α, c-Jun N-terminal kinase (JNK), and extracellular signal-regulated kinase (ERK)2, which are related to upregulation of proinflammatory cytokines, including IL-6 (30). The phosphorylated levels of p38, JNK, and ERK1/2, their active form, has been shown to be increased by TcdB in EGCs *in vitro* (20). Thus, p38, JNK, and ERK1/2 signaling might be involved in the regulation of *IL-6* expression by A2A on EGCs challenged with *C. difficile* toxins.

Our work also demonstrated that blocking A2B receptor in EGCs increased *IL-6* expression induced by *C. difficile* toxins. However, our *in vivo* data using a A2BKO mice showed that deletion of A2B during CDI resulted in decreased IL-6 in cecum. Similarly, A2B antagonist has not been shown to affect the levels of secreted IL-1β in EGCs induced by LPS and palmitate *in vitro* (12). On the other hand, in mice challenged with TcdA, A2B antagonist decreased IL-6 synthesis in colon tissues (31). A similar effect was observed in a pre-clinical model of CDI (26) and in Carrageenan-induced edema (46). These findings suggest that in the presence of an intestinal insult *in vivo*, A2B has mostly pro-inflammatory effect in epithelial and immune cells likely masking its partial anti-inflammatory effect in EGCs. In addition, because A2B blockage promoted both an anti-apoptotic effect and increased IL-6 expression in EGCs challenged with *C. difficile* toxins, IL-6 might contribute to the antiapoptotic effect of A2B inhibition in EGCs. IL-6, as a pleiotropic cytokine, has been shown to promote survival, inhibiting the apoptosis of neuronal cells and other cells (47–50).

Another important finding was that A2A activation or A2B blockage in EGCs challenged by *C. difficile* toxins reduced the upregulation of S100B. *In vivo*, A2B deletion also reduced cecal S100B levels. EGCs are the main source of S100B in the gut. In inflammatory conditions, high levels of S100B is released and is associated with increased inflammatory response during CDI (5) and other inflammatory conditions (15). Increased S100B has also been observed by others to be associated with cell death (15, 28). Here, we also showed that infected A2BKO mice exhibited lesser enteric neuron loss compared to WT infected mice. How S100B participates in EGCs death induced by *C. difficile* toxins remains unclear.

EGCs can play a dual role in homeostasis and inflammatory conditions. Under physiological conditions, EGCs interact with other cells to maintain the integrity of epithelial barrier and regulate secretion and motility (13, 51). However, in inflammatory conditions EGCs are activated and produce proinflammatory mediators, such as S100B, which can act in other cells and EGCs to potentiate the inflammatory response (5, 15). Ablation of EGCs has been shown to result in fulminant enteritis (52). Interventions to prevent EGC death and regulate their inflammatory response may improve outcomes of CDI. Although the role of adenosine receptor signaling in EGCs challenged with *C. difficile* toxins seems to be complex, upregulation of A2A and downregulation of A2B in EGCs appear to be protective mechanisms against the deleterious effects of these toxins (**Figure 6E**). A2B activation appears to be critical in inducing apoptosis in the presence of TcdA or TcdB and thus, presents as a potential target of intervention to prevent or diminish injury in the enteric nervous system during CDI.

## Data availability statement

The datasets presented in this study can be found in online repositories. The names of the repository/repositories and accession number(s) can be found in the article/**Supplementary Material**.

## Ethics statement

The animal study was reviewed and approved by Committee on the Ethics of Animal Experiments of the University of Virginia (Protocol number: 4096).

## Author contributions

DC wrote the manuscript, performed all the experiments, analyzed and organized the data. JS, SG, DB, FM, AL and MJ helped in the acquisition of data. GB reviewed the manuscript. CW contributed to the organization of the manuscript, reviewed, and approved the final version. All authors contributed to the article and approved the submitted version.
