# Supplementary material for: Adenosine receptors differentially mediate enteric glial cell death induced by Clostridioides difficile Toxins A and B
Source: Front Immunol. 2023 Jan 16;13:956326. doi: 10.3389/fimmu.2022.956326 (PMC9885079; doi:10.3389/fimmu.2022.956326)
Supplement: Supplementary file 1 [file DataSheet_1.docx]

**Supplementary Figure 1**

**
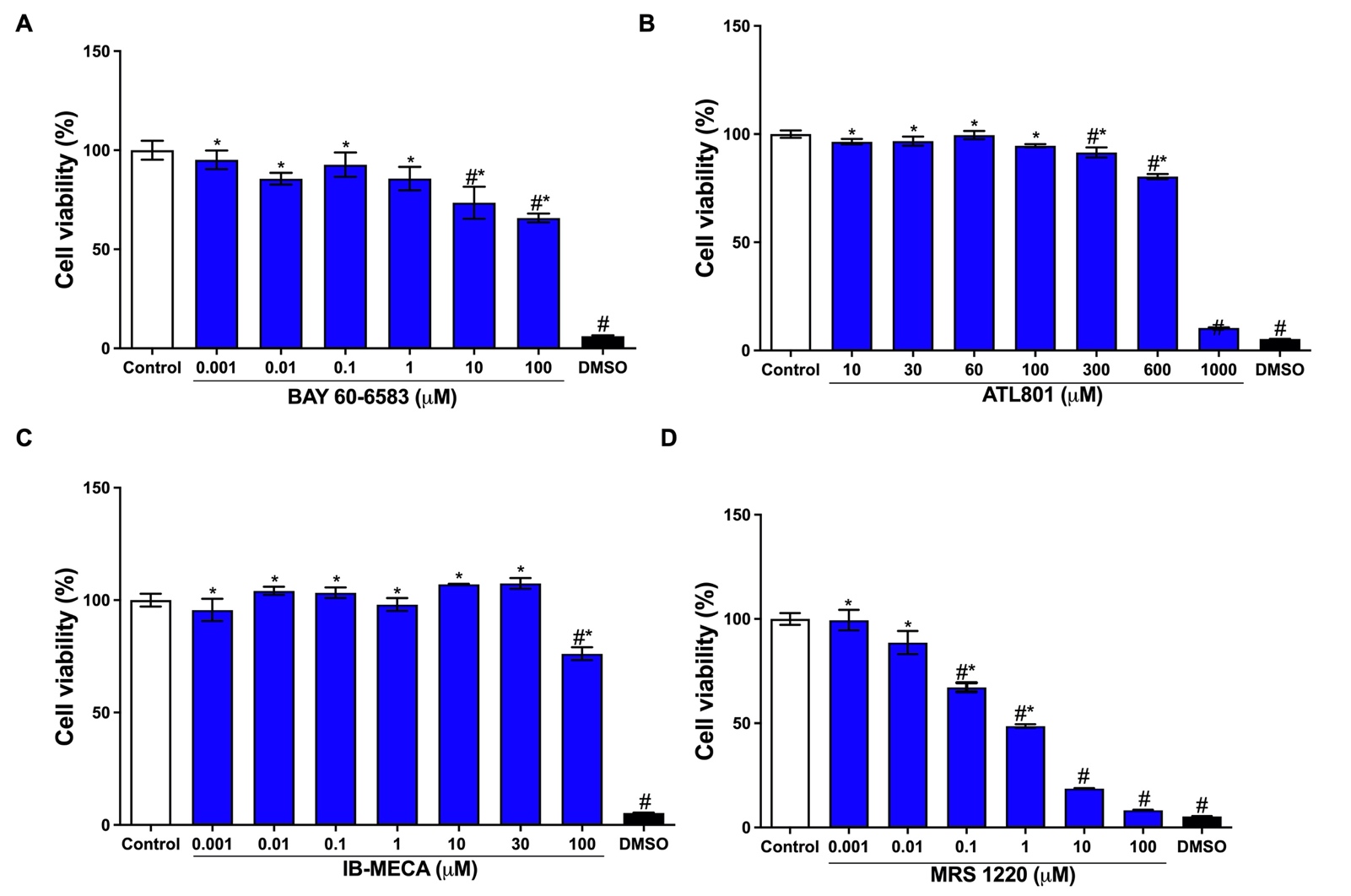
**

**Effects of modulators of adenosine receptors on EGCs viability.** EGCs viability were assessed by MTT assay after 18h incubation with Bay60-6583 (A), ATL801 (B), IB-MECA (C) and MRS1220 (D). DMSO was used as a death control group. Data are presented as the mean ± s.e.m. (*n* = 6). #p<0.0001 versus control group and * p<0.0001 versus DMSO group. One-way ANOVA followed by Tukey test.

**Supplementary Figure 2**

**
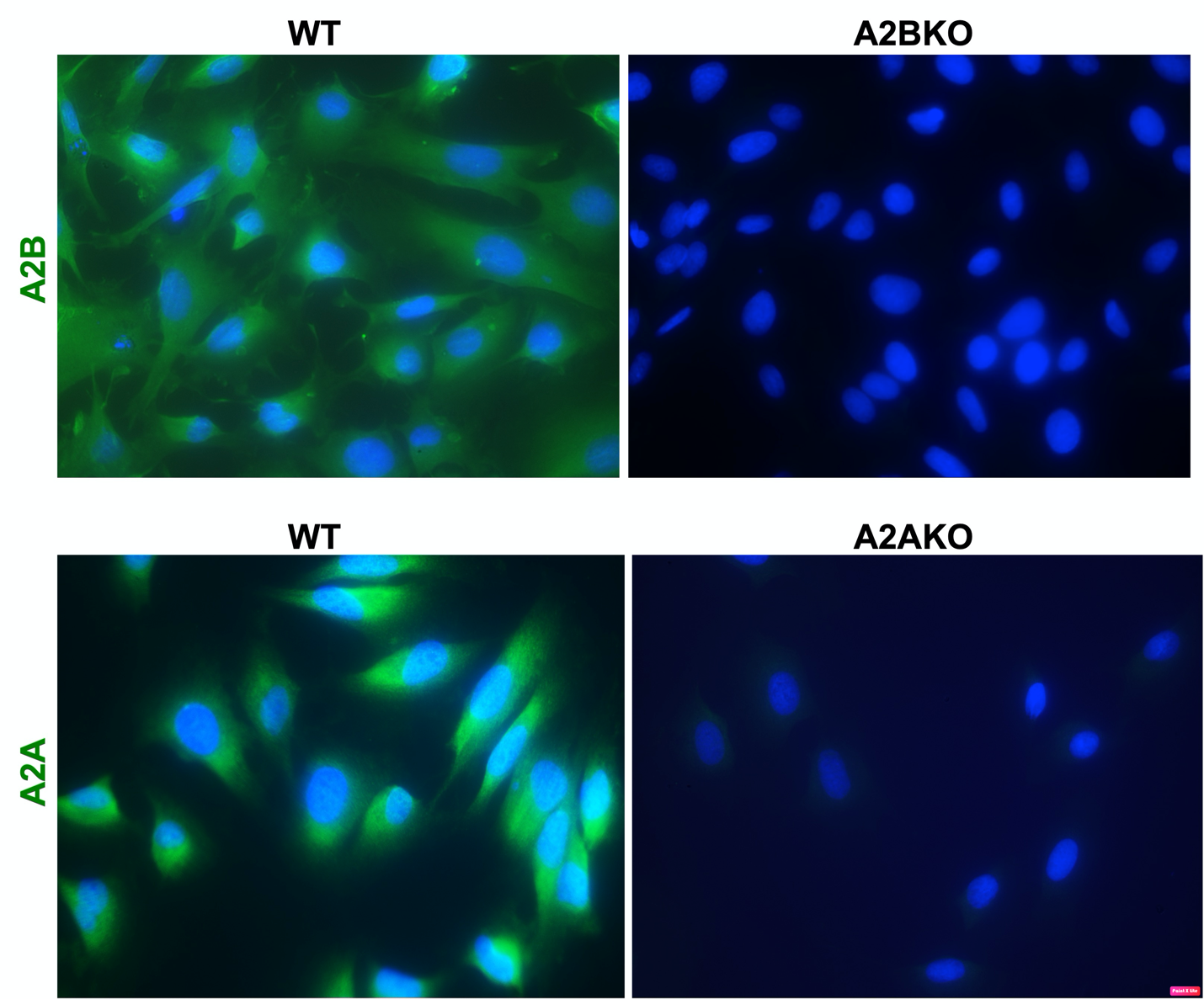
**

Immunofluorescence images of *A2A* and *A2B* immunostaining (green) and DAPI (blue) as a nuclear staining in Wild-type EGC (WT) and EGC post-knockdown of A2A (A2AKO) or A2B (A2BKO) using a lentiviral-mediated CRISPR/cas9 genome editing.

**Supplementary Figure 3**

**
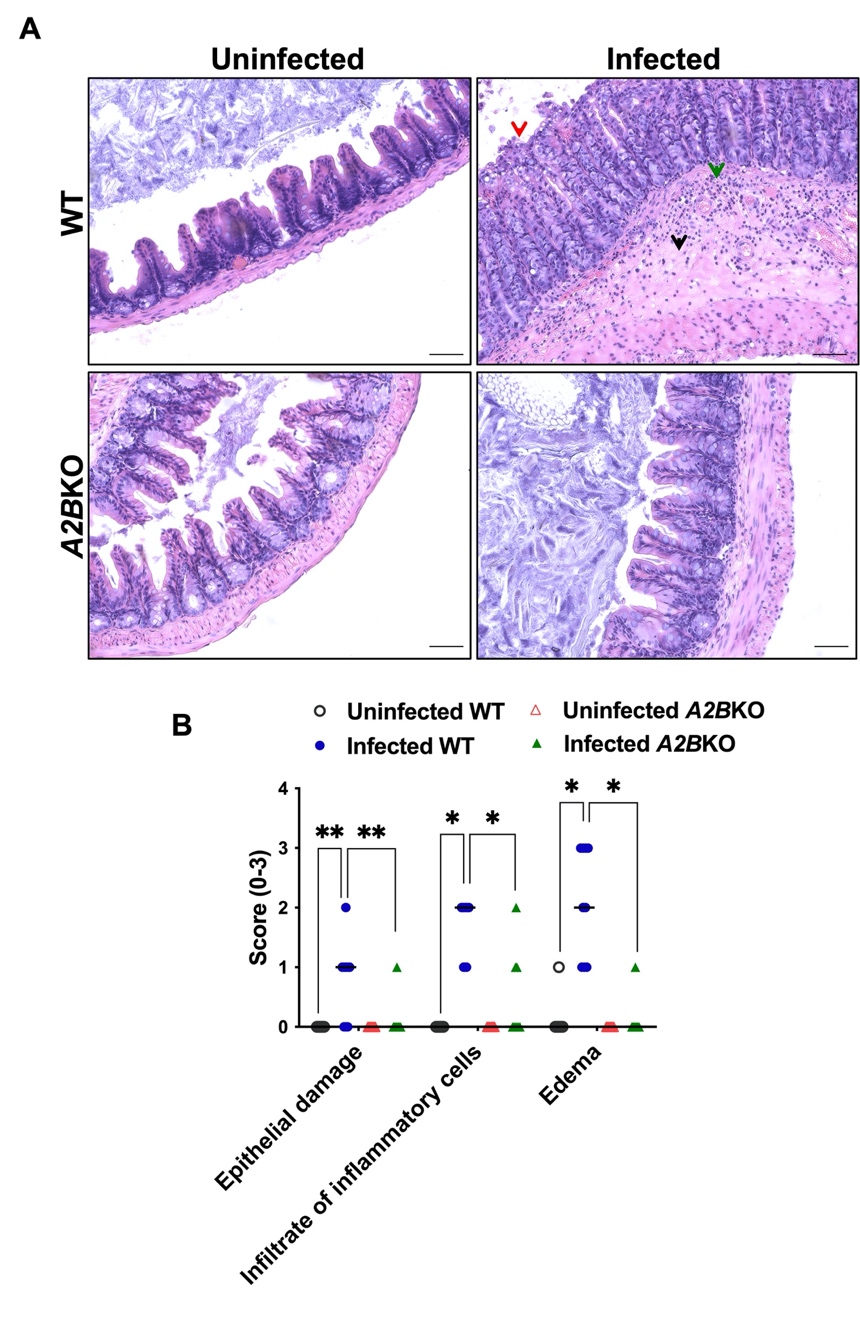
**

(A) Representative H&E stains of cecum tissues collected from uninfected and *C. difficile* infected mice (WT and *A2B* knockout-*A2B*KO) at day 3 post-infection (p.i.). CDI promotes damage of cecal epithelium (red arrow), edema (black arrow) and cell infiltrate (green arrow). Scale bars, 100 µm. (B) Histopathologic score (median, 0-no damage, and 3-intense damage) of the epithelial damage, infiltration of inflammatory cells, and submucosal edema. Kruskal-Wallis nonparametric test followed by Dunn’s test. **p=0.02; *p<0.0001.

**Supplementary Figure 4**

**
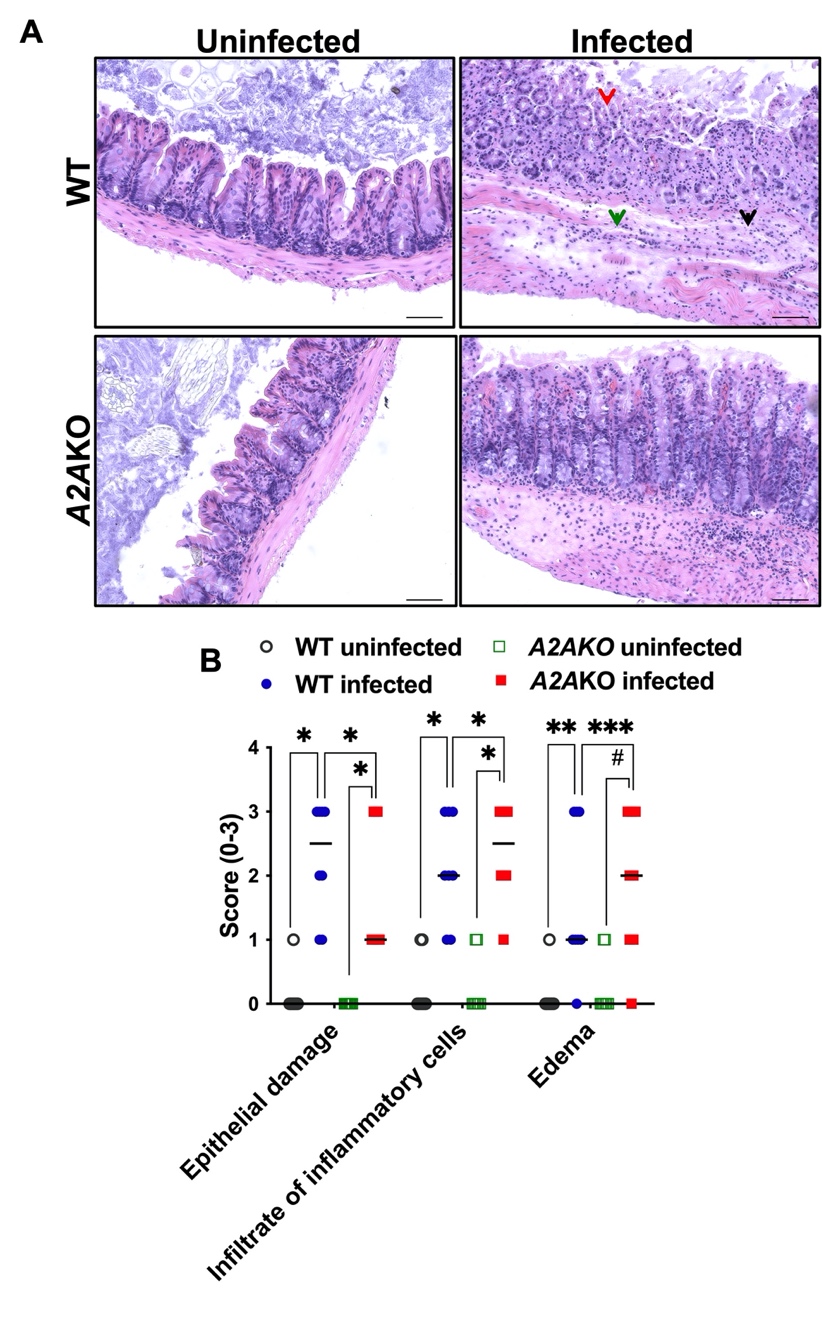
**

(A) Representative H&E stains of cecum tissues collected from uninfected and *C. difficile* infected mice (WT and *A2A* knockout-*A2A*KO) at day 3 post-infection (p.i.). CDI promotes damage of cecal epithelium (red arrow), edema (black arrow) and cell infiltrate (green arrow). Scale bars, 50 µm. (B) Histopathologic score (median, 0-no damage, and 3-intense damage) of the epithelial damage, infiltration of inflammatory cells, and submucosal edema. Kruskal-Wallis nonparametric test followed by Dunn’s test. *p<0.0001; **p=0.002; ***p=0.001; #p=0.0003.

**Supplementary Figure 5**

**

**

Levels of *A1* gene expression (mean ± s.e.m) by qPCR in enteroglial cell (EGC/PK060399) challenged with TcdA and TcdB (n=6). Two-way ANOVA followed by Turkey test was used.

**Supplementary Figure 6**

**
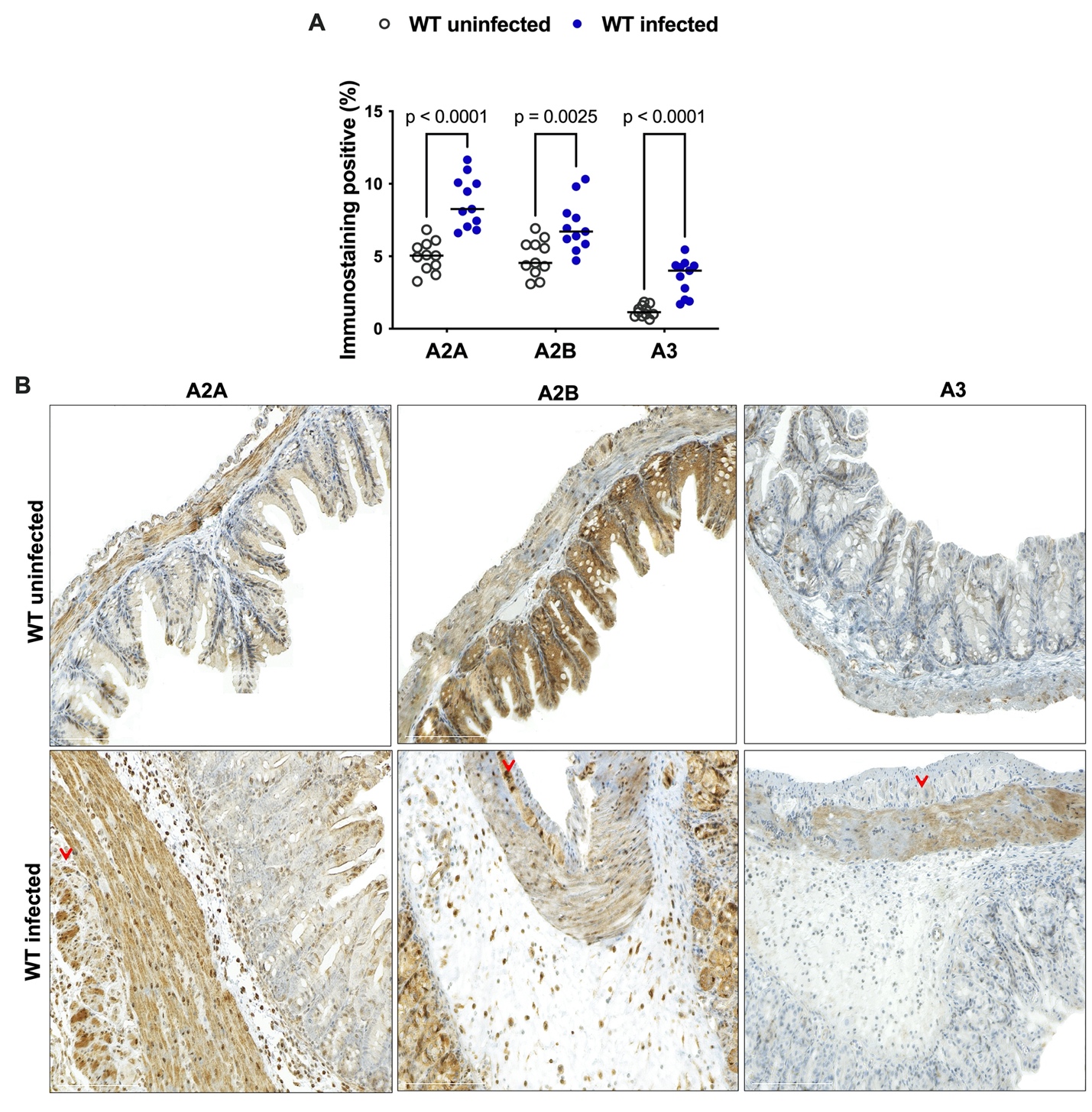
**

**Adenosine receptors (A2A, A2B and A3) distribution in cecum samples during *C. difficile* infection.** (A) Analysis of A2A, A2B and A3 immunostaining in cecum samples from WT uninfected and infected mice on day 3 post *C. difficile* infection (n=10-11). Mann-Whitney’s test was used. (B) Representative photomicrographs of A2A, A2B and A3 immunostaining in cecum samples from WT uninfected and infected mice on day 3 post *C. difficile* infection. The red arrow shows a positive immunostaining of A2A, A2B and A3 in the myenteric plexus.

**Supplementary Figure 7**

**
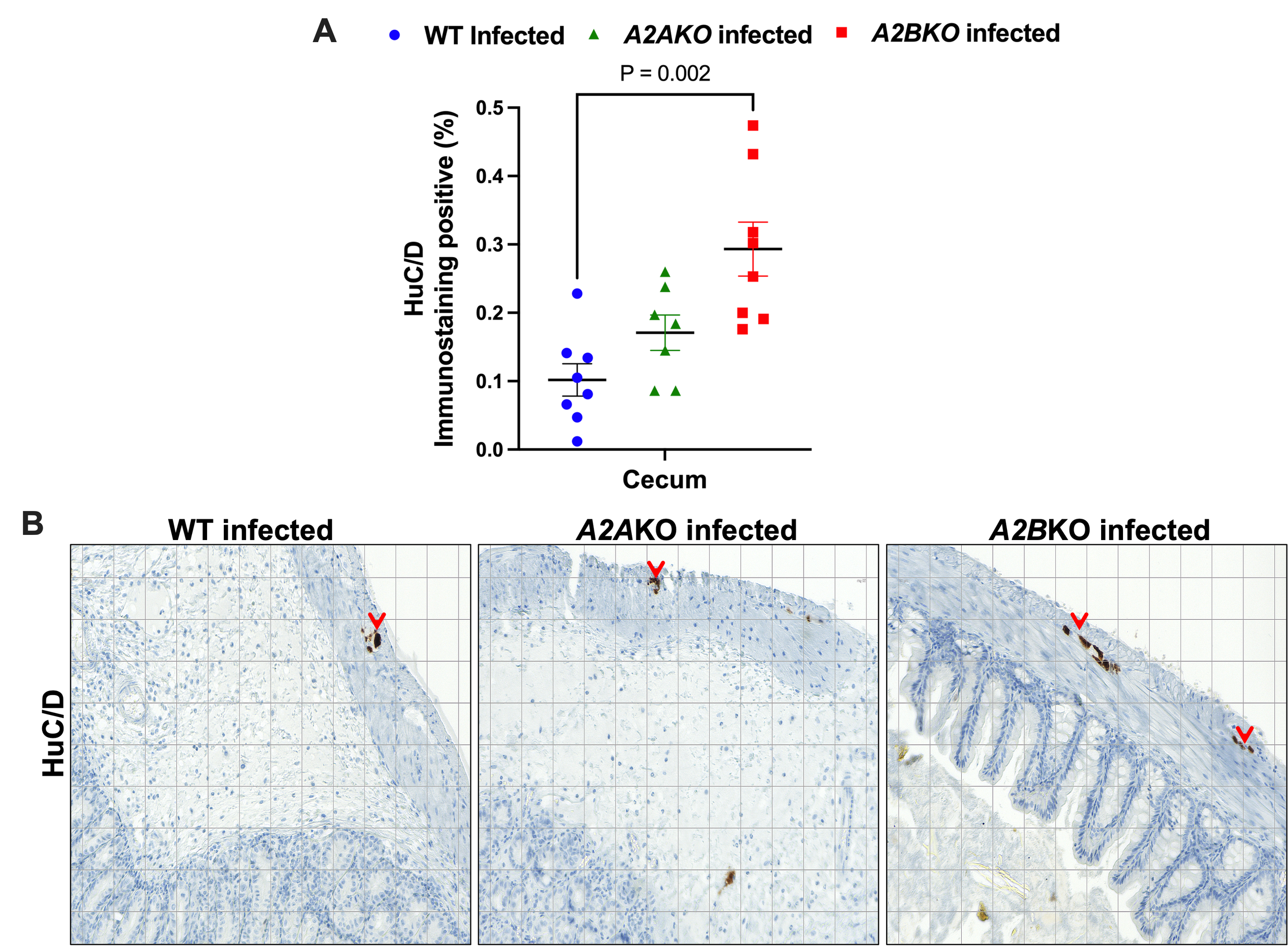
**

**Deletion of *A2B*, but not *A2A*, increases HuC/D in cecum samples during *C. difficile* infection.** (A) Analysis of HuC/D (a marker of enteric neuron) immunostaining in cecum samples from WT, *A2*AKO and *A2*BKO mice on day 3 post *C. difficile* infection (n=8-9). Kruskal-Walli’s test followed by Dunn’s was used. (B) Representative photomicrographs of HuC/D (red arrow) immunostaining in cecum samples from WT, *A2*AKO and *A2*BKO mice on day 3 post *C. difficile* infection.

**Supplementary Figure 8**


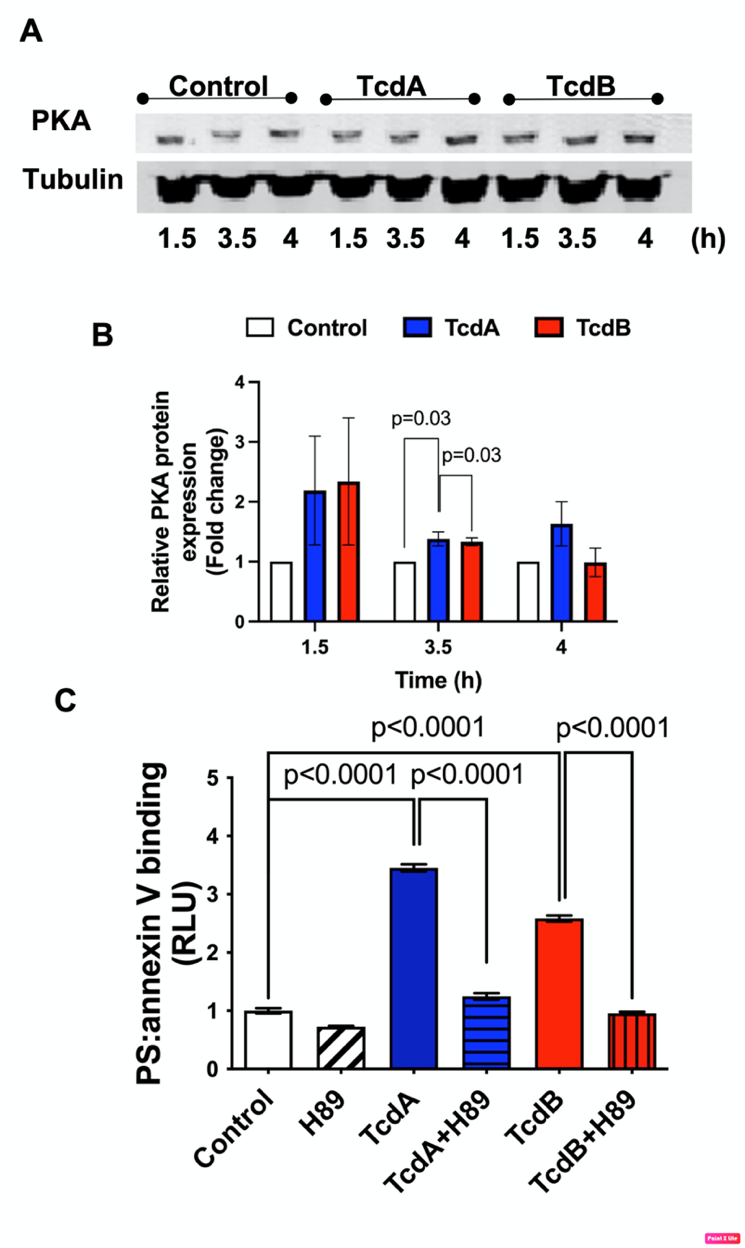


(A) Western blot (WB) bands of PKA and tubulin in enteroglial cell (EGC/PK060399) exposed to TcdA and TcdB at 1.5, 3.5 and 4 h of incubation. (B) WB analysis of PKA in enteroglial cell (EGC/PK060399) exposed to TcdA and TcdB at 1.5, 3.5 and 4 h of incubation. Tubulin was used to normalize the levels of PKA. (C) Cell death analyzed by RealTime-Glo annexin V apoptosis assay (mean ± s.e.m, n=6) in enteroglial cell (EGC/PK060399) challenged with TcdA and TcdB for 18 h in the presence and/or absence of 10 µM H89 (a PKA inhibitor), added one hour prior to *C. difficile* toxin challenge. One-way ANOVA followed by Turkey test was used.

**Supplementary Figure 9**

**

**

WB analysis of pCREB in enteroglial cell (EGC/PK060399) exposed to TcdA and TcdB at 1.5, 3.5 and 4 h of incubation. Tubulin was used to normalize the levels of pCREB.

**Supplementary Figure 10**

**
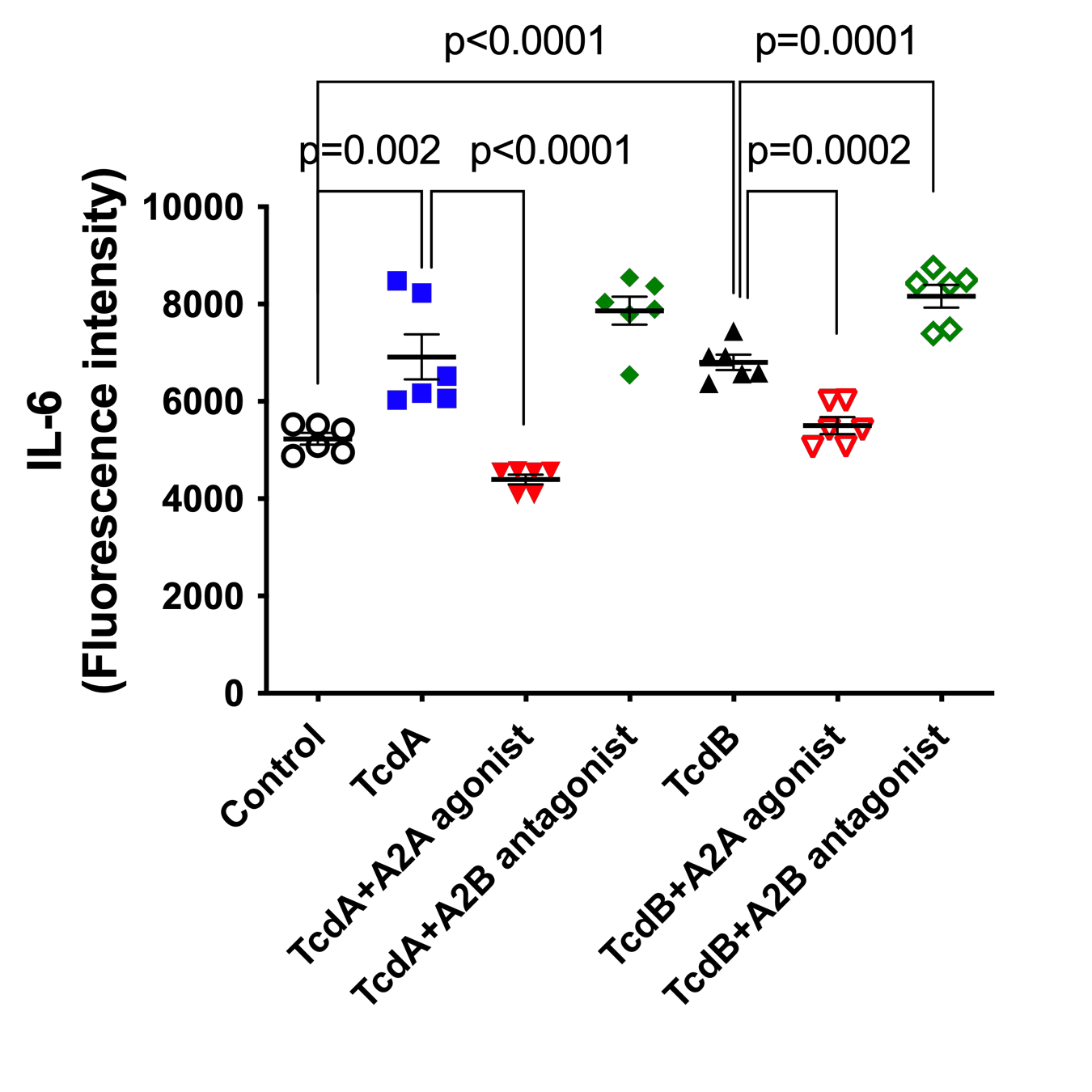
**

**A2A agonist, but not A2B antagonist, decreases TcdA- and TcdB-induced increase on IL-6 levels in EGCs.** Fluorescence intensity of IL-6 immunostaining measured by ImageJ software in EGCs incubated for 18h with TcdA or TcdB and A2A agonist (ATL313, 300µM) or A2B antagonist (ATL801, 100µM) 1h prior to toxin challenge. The data are the mean ± SEM. One-way ANOVA followed by the Tukey test was used. p value is represented in the graph.

**Supplementary Figure 11**

**
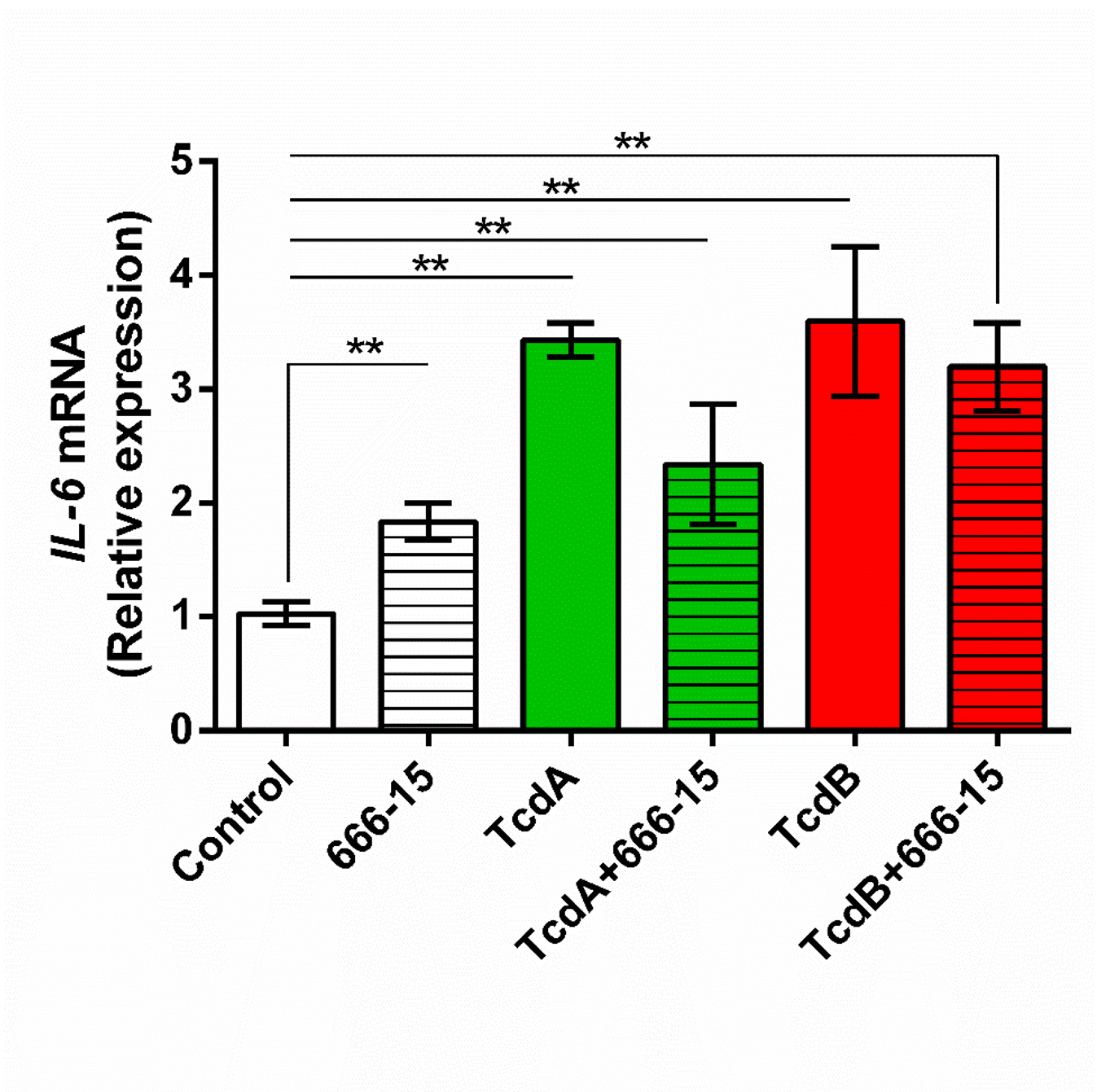
**

Analysis of *IL-6* gene expression (mean ± s.e.m) by qPCR in enteroglial cell (EGC/PK060399) challenged with TcdA and TcdB for 18h in the presence or absence of 0.5 µM 666-15 (a CREB inhibitor). These experiments are from two different replicates. One-way ANOVA followed by Turkey test was used. **p<0.0001.

**Supplementary Figure 12**

**
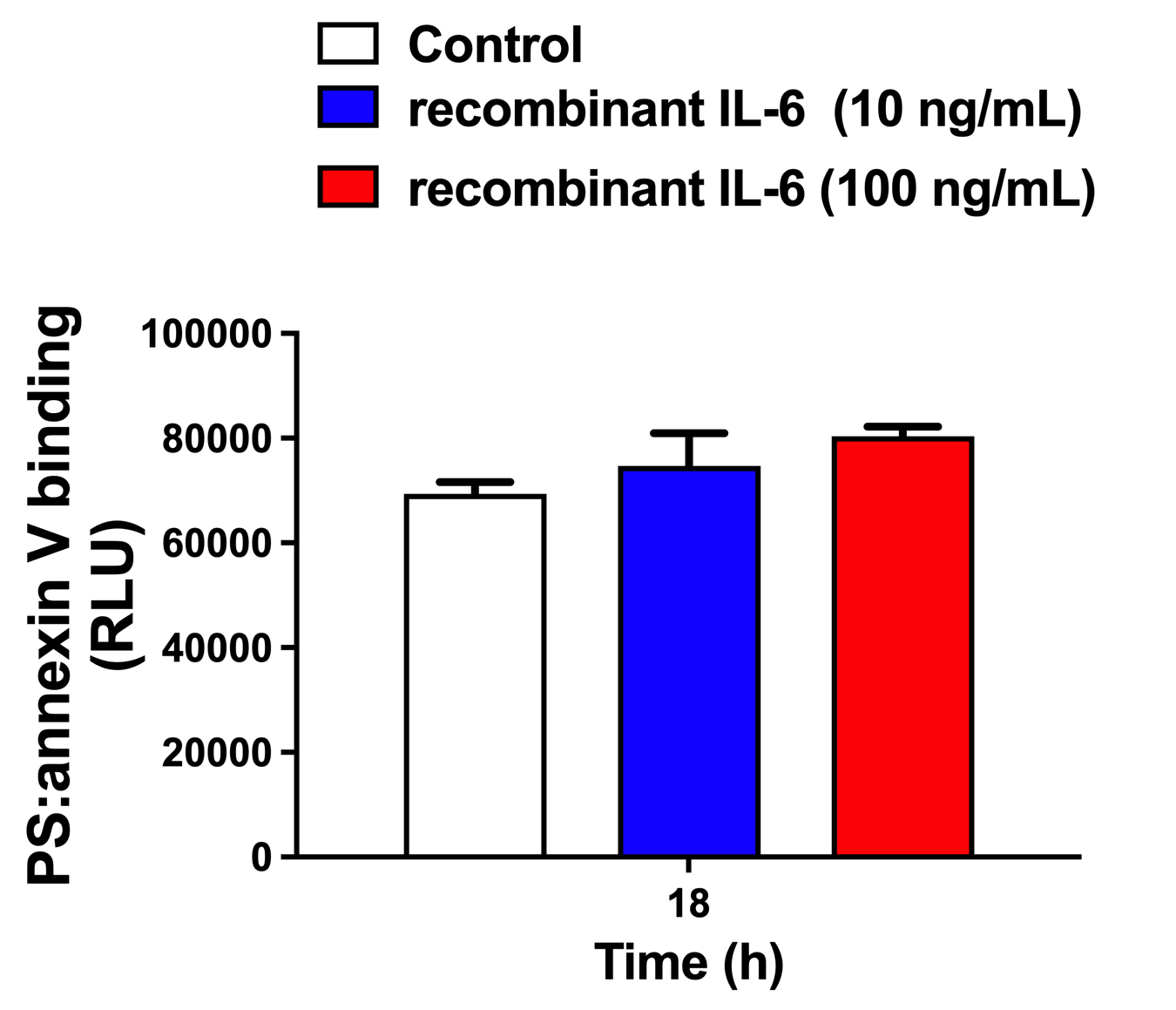
**

Cell death analyzed by RealTime-Glo annexin V apoptosis assay (mean ± s.e.m, n=6) in enteroglial cell (EGC/PK060399) in the presence and/or absence of 10 ng/ mL or 100 ng/mL recombinant IL-6. One-way ANOVA followed by Turkey test was used.

**Supplementary Figure 13**

**
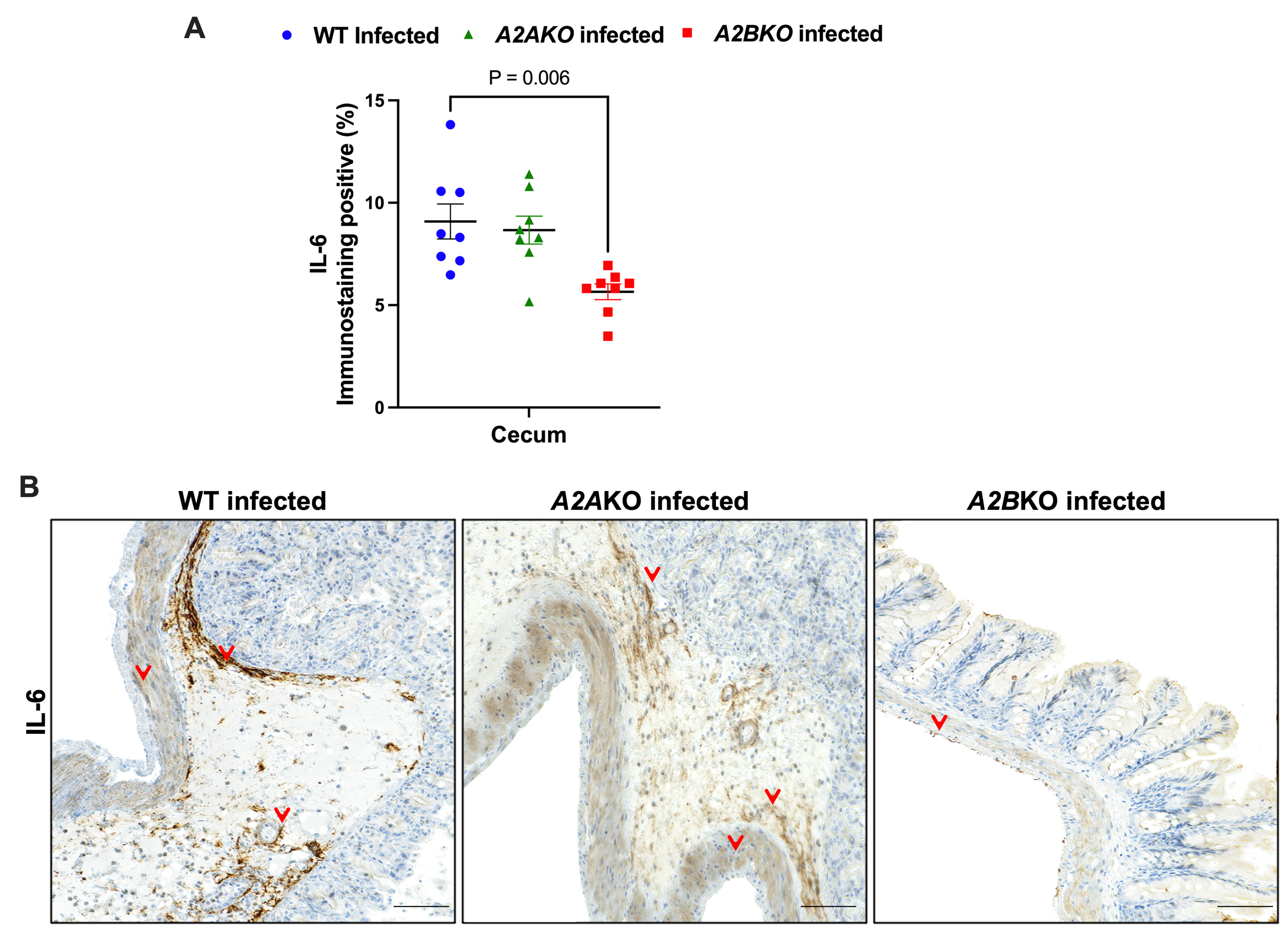
**

**Deletion of *A2B*, but not *A2A*, decreases IL-6 in cecum samples during *C. difficile* infection.** (A) Analysis of IL-6 immunostaining in cecum samples from WT, *A2*AKO and *A2*BKO mice on day 3 post *C. difficile* infection (n=8-9). Kruskal-Walli’s test followed by Dunn’s was used. (B) Representative photomicrographs of IL-6 (red arrow) immunostaining in cecum samples from WT, *A2*AKO and *A2*BKO mice on day 3 post *C. difficile* infection.

**Supplementary table 1. Primers used in this paper**

| Primers | Primers  Direction | Nucleotide Sequence (5’-3’) |
| --- | --- | --- |
| *GAPDH* | Forward  Reverse | AGACAGCCGCATCTTCTTGT  CTTGCCGTCGGTAGAGTCAT |
| *A1R* | Forward  Reverse | ATAGCTGGCTGCTGGATTCTC  CACTCAGGTTGTTCCAGCCA |
| *A3R* | Forward  Reverse | TGGTCTTCACCCATGCTTCC    AGCTTGACTCGCAGGTATCG |
| *A2AR* | Forward  Reverse | GTCCTCACGCAGAGTTCC  AAGCCATTGTACCGGAGTGG |
| *A2BR* | Forward  Reverse | GTGGCTGTCGAGCGGTATC  GCTCGTGTTCCAGTGACCAA |
| *IL-6* | Forward  Reverse | GCCAGAGTCATTCAGAGCAAT    GTTGGATGGTCTTGGTCCTTA |
| *S100B* | Forward  Reverse | TTCAGGGAGAGAGGGTGACAA  CTTCCTGCTCTTTGATTTCCTCC |
